# Massive scabies outbreak in Rohingya refugee camps, Cox’s Bazar: Epidemiology and impact of a mass drug administration (MDA) campaign – A retrospective study

**DOI:** 10.1371/journal.pntd.0013832

**Published:** 2026-07-01

**Authors:** Charls Erik Halder, Md Abeed Hasan, James Charles Okello, David Otieno, Emmanuel Roba Soma, Partha Pratim Das, Md. Mostafizur Rahman, Md. Atiquzzaman, Hamim Tassdik, Dickson Wafula Barasa, Julekha Tabassum Poly, Abdullah Al Mamun, Abu Toha Md. Rezuanul Haque Bhuiyan, U. Maung Prue

**Affiliations:** 1 Migration Health Division, International Organization for Migration (IOM), Dhaka, Bangladesh; 2 Epidemiology Team, World Health Organization (WHO), Dhaka, Bangladesh; 3 Office of the Refugee Relief and Repatriation Commissioner (RRRC), Cox’s Bazar, Bangladesh; University of Notre Dame, UNITED STATES OF AMERICA

## Abstract

**Background:**

Since 2021, Rohingya refugee camps in Cox’s Bazar, Bangladesh, have experienced a significant and concerning increase in the prevalence of scabies. In response to the massive outbreak, an extensive Mass Drug Administration (MDA) campaign was conducted by WHO Bangladesh, in collaboration with the Government of Bangladesh and Health Sector partners, from November 29, 2023, to February 01, 2024.

**Objectives:**

The objectives of the study were: a) to determine the epidemiological characteristics (i.e., magnitude, age-sex distribution, and attack rate) of the scabies outbreak in the Rohingya refugee camps; and b) to evaluate the impact and durability of MDA in reducing the burden of scabies in the refugee camps.

**Methodology:**

This was a retrospective observational study that utilized deidentified and anonymized data from 35 health facilities of the International Organization for Migration (IOM) spanning the period from January 1, 2021, to December 31, 2024.

**Results:**

A total of 384,852 cases of scabies were reported, with an overall attack rate of 5,562.59 scabies cases per 10,000 population over 4 years. Females had a slightly higher case proportion (53.51%) and attack rate (ARR: 1.104, p < 0.001). Children under 5 years (36.31%) had the highest burden and attack rate, about twice the overall attack rate. Using this age group as reference, the attack rate declined significantly with increasing age, 5,561.85 among adolescents (ARR: 0.496, p < 0.001) to 4,377.88 among individuals over 60 years (ARR: 0.390, p < 0.001). 77% of the cases were reported among the Rohingya refugees; however, the attack rate was higher among host communities (7,721.34 per 10,000 vs. 5,138.24 per 10,000 among refugees). Overall, 5.80% of scabies cases were associated with secondary bacterial infections. Interrupted time series (ITS) analysis showed a sharp and immediate decline in scabies cases following MDA initiation, representing a reduction of 1,885 cases per week (p < 0.001) – equivalent to a weekly decline of approximately 100% from the pre-MDA peak. The decline persisted for 6 months (decreasing trend of 69.98 cases/week, p < 0.001), after which a statistically significant upward rebound was observed (72.04 additional cases per week, p = 0.002).

**Conclusion:**

The study revealed a high burden of scabies among the Rohingya refugees and the adjacent host community. MDA was an effective approach for a rapid and substantial reduction of the disease burden. However, the impact cannot be sustained unless the underlying factors of the scabies outbreak are addressed.

## Author summary

Scabies is a parasitic skin disease that disproportionately affects people living in overcrowded and resource-limited settings. In the Rohingya refugee camps in Cox’s Bazar, Bangladesh, one of the world’s largest and most protracted humanitarian crises, we documented a massive scabies outbreak involving nearly 385,000 reported cases between 2021 and 2024. We found that children under five years and females carried the highest disease burden, and that the attack rate was higher in the adjacent host community than among the Rohingya refugees. In response to the outbreak, a large-scale mass drug administration (MDA) campaign using oral ivermectin was implemented in late 2023 and early 2024, reaching approximately one million individuals across 33 camps. Our interrupted time series analysis demonstrated that MDA produced a sharp and immediate reduction in weekly scabies cases. However, this effect was not sustained beyond six months, with cases rebounding significantly thereafter. These findings highlight the critical role of MDA in rapidly controlling scabies in high-burden humanitarian settings, while underscoring that sustained control requires addressing the underlying structural factors – particularly overcrowding and inadequate living conditions — that drive ongoing transmission.

## Introduction

Scabies, classified among neglected tropical diseases, impacts over 400 million individuals annually [1,2]. It is a parasitic skin disease caused by the *Sarcoptes scabiei* mite, which burrows into the skin, lays eggs, and incites severe pruritus [3,4]. Scabies is primarily transmitted through direct skin-to-skin contact [3,4]. Secondary bacterial skin infections from scabies are a significant risk factor for immune-mediated complications, including acute post-streptococcal glomerulonephritis (manifesting as kidney disease), rheumatic heart disease, and death [2,3,5,6]. While scabies is endemic globally, its prevalence is notably higher in hot and tropical countries as well as in regions characterized by high population density [2]. Consequently, refugees and displaced communities face an elevated risk of transmission due to overcrowded living conditions that facilitate close skin-to-skin contact, as well as broader constraints in living conditions, challenges in maintaining personal hygiene and laundering clothes and bedding, limited access to healthcare, shortage of scabicidal drugs, and socio-cultural factors such as language barriers and stigma [5,7–9]. A high burden of scabies has been reported in various refugee settings across the globe, such as Greece (with a proportional morbidity 5.6%) and Duhok, Iraq (with upto 20% prevalence), highlighting the global burden of the disease in the humanitarian contexts [7,10].

Following decades of persecution and repeated displacement, approximately 939,344 Rohingya refugees fled to Bangladesh in August 2017 [11]. As of December 31, 2023, they reside in densely populated camps in Cox’s Bazar, where overcrowding, fragile shelters, poor water, sanitation, and hygiene(WASH) infrastructure, and harsh monsoon conditions significantly increase vulnerability to infectious disease outbreaks [11]. The camps have experienced multiple outbreaks or surges of infectious diseases, including diphtheria, measles, COVID-19, and acute watery diarrhea (AWD)/cholera [12–16]. Since 2021, a significant and concerning surge in the prevalence of scabies has been observed in the refugee camps. The high burden of scabies has led to an increased demand for heightened medical attention and additional resources, exacerbating financial strain to an already strained health system within this protracted humanitarian crisis. Simultaneously, it contributes to disruptions in daily life, potentially hindering social interactions and economic activities within the refugee community.

In response to the massive outbreak of scabies, from November 29, 2023, to February 01, 2024, an extensive Mass Drug Administration (MDA) campaign was conducted targeting nearly 1 million beneficiaries residing in 33 camps in Cox’s Bazar and Bhasan Char Island in Bangladesh. This campaign was recognized as the world’s most extensive MDA campaign for scabies treatment and prevention [1,2]. MDA is a public health strategy that involves providing treatment to entire population, irrespective of their disease status, and has been practiced extensively in recent decades as part of a global effort to control and eliminate neglected tropical diseases [17]. For the MDA campaign in Cox’s Bazar, Ivermectin 3mg tablets and Permethrin 5% cream (for contraindicated cases) were used.

Despite scabies being a critical public health concern in refugee and displacement populations - due to its association with disability, stigma, and exacerbation of poverty, there are very limited studies available describing the epidemiology and management of scabies in these settings [10,18–20]. Understanding the epidemiology of scabies, including its patterns and magnitudes of outbreaks, is essential for effective public health planning, prevention, and response.

A systematic review and meta-analysis of 11 reports on the impact of MDA found it to be a highly effective strategy in reducing the prevalence of scabies [21]. However, the study concluded that further research is warranted to understand the effectiveness of MDA regimens in larger populations and to determine the durability of impact. Therefore, in addition to explaining the epidemiology and magnitude of the outbreak, our study evaluated the impact of the MDA campaign and its durability.

The objectives of the study were: a) to determine the epidemiological characteristics of the scabies outbreak in the Rohingya refugee camps, including magnitude, age-sex distribution, and attack rate; and b) to evaluate the impact and durability of MDA in reducing the burden of scabies in the refugee camps. The findings of the research will assist health programmes and policymakers in predicting the scale and characteristics of scabies outbreaks, as well as the effectiveness of public health interventions, thereby facilitating proper planning for the effective integration of scabies prevention and management within the essential health service model in Bangladesh and similar humanitarian and refugee settings globally.

## Methodology

### Ethics statement

The research team received ethical clearance (CoxMC/2023/017) from the Ethical Review Board, Cox’s Bazar Medical College Hospital. The development of the research was discussed with, and necessary clearance was obtained in coordination with the Office of the Civil Surgeon and the Refugee Relief and Repatriation Commissioner (RRRC) Health Coordinator, who oversee health activities within the Rohingya refugee response in Cox’s Bazar, Bangladesh. The MDA campaign evaluated in this study was implemented as a public health response by WHO Bangladesh, following formal approval from the Office of the Civil Surgeon, Government of Bangladesh, and after obtaining technical clearance from the WHO Country Office and WHO SEARO. Internal programmatic authorization was also obtained from the IOM Migration Health Division Research Unit, both for the study and retrospective programme data use for the study. As the analysis was conducted on anonymized, aggregated programmatic data generated during outbreak response activities, and not as a prospective research intervention, individual-level informed consent was not required.

### Study design

This was a retrospective observational study that used deidentified and anonymized data from the International Organization for Migration’s (IOM) scabies patient database.

### Study site and population

The study was implemented in Rohingya refugee camps in Ukhiya and Teknaf in Cox’s Bazar. The study population included all individuals who were clinically diagnosed with scabies and received care at any of the 35 IOM-supported health facilities in Ukhiya and Teknaf, Bangladesh, between 1st January 2021 and 31st December 2024.

### Data collection

Deidentified and anonymized aggregated data pertaining to scabies cases were retrieved from the IOM Cox’s Bazar’s health information management record spanning the period from 2021 to 2024. The data included age, sex, camp location, and diagnosis of secondary infection. All data were collected using the IOM Cox’s Bazar OPD form, which was further transmitted electronically using Kobo Toolbox and synchronized at the central database. The data were verified and cleaned daily at the central level before being exported to an Excel spreadsheet for analysis. Weekly scabies case counts were defined as the total number of clinically diagnosed scabies cases aggregated per epidemiological week across all included facilities. Infected scabies cases were defined as scabies cases presenting with clinical signs of secondary bacterial infection.

### Scabies diagnosis and clinical management

All scabies cases were clinically diagnosed at 35 IOM-supported facilities in Ukhiya and Teknaf. Médecins Sans Frontières (MSF) clinical guidelines [22] – a diagnosis and treatment manual was used for diagnosis and clinical management of the cases. Scabies was diagnosed based on the presence of intense pruritus, typically worse at night, accompanied by characteristic skin lesions—including papules, burrows, or vesicles—in typical distribution sites such as the interdigital spaces, wrists, flexor surfaces of the elbows, axillae, and the periumbilical area in adults, and the face, scalp, and palms in infants and young children. Clinical diagnosis was further supported where applicable by evidence of similar symptoms in close household contacts [23]. Secondary bacterial infection (infected scabies) was recorded when the treating clinician identified clinical features consistent with superimposed bacterial skin infection, including the presence of pustules, crusting, oozing, or impetiginization at scabies lesion sites, as assessed during routine clinical encounter. Topical 5% permethrin cream was mainly used for the treatment of uncomplicated scabies and oral ivermectin and benzyl benzoate were used selectively based on clinical indication and availability.

### Mass Drug Administration (MDA Campaign)

Between November 29, 2023, and February 01, 2024, WHO Bangladesh, in collaboration with the Government of Bangladesh and Health Sector partners, implemented the world’s largest Ivermectin-based MDA campaign in response to the massive scabies outbreak among the Rohingya refugees in 33 camps across Cox’s Bazar and Bhasan Char [24]. The initiative was triggered by a WHO prevalence survey in May 2023, which reported a scabies prevalence of 39% among Rohingya refugees, exceeding the 10% threshold for mass drug administration [24].

The campaign targeted approximately 992,500 individuals across 33 refugee camps in Ukhiya, Teknaf, and Bhasan Char. The target population (denominator) for the MDA campaign was derived from camp-level population estimates maintained by UNHCR, representing the total population residing within the selected camps during the intervention period [24].

Following the WHO recommendations for scabies control, the MDA regimen consisted of two doses of oral ivermectin at 200 µg/kg body weight, with an interval of 7–14 days between doses. Since, ivermectin is not recommended in children less than 15 kg or 90 cm in height, pregnant women in the first trimester, and those who have the known allergy to ivermectin, 5% permethrin cream was provided for individuals with contraindications to ivermectin, including young children and pregnant women [2].

The campaign was delivered using a Directly Observed Treatment (DOT) strategy, whereby trained health workers ensured that treatment was administered appropriately at the community level. Prior to implementation, WHO trained 100 healthcare workers, 130 community health worker supervisors, and over 1,600 community health workers trained to support the delivery of MDA [24].

To improve coordination, WHO established the MDA Implementation Advisory Committee, comprising members from both the Health and WASH Sectors, to supervise implementation and finalise the micro-plan for MDA. Furthermore, 68 storage locations—including 48 health facilities—were designated to ensure secure management, storage, and distribution of drugs prior to the campaign [24].

The campaign provided medications to over 992,500 Rohingya refugees, including 5.2 million Ivermectin tablets and 185,000 tubes of Permethrin cream. IOM contributed 60,000 drugs (Ivermectin, 6 mg) in this campaign, allowing the second dosing of the drug among 33,970 residents in Camp 09 and Camp 20 Extension [24]. The coverage of the campaign was exceptionally high, exceeding 99% of the target for both doses [24,25].

### Statistical analysis

Analysis of epidemiological characteristics: The cleaned dataset was summarized into scabies cases and infected scabies by weeks, months, and years through descriptive statistics. Attack rates were calculated annually and overall, using total scabies cases divided by the respective catchment population, expressed per 10,000 person-years. Attack rates were also segregated for years, age, sex, geographical location (camps and Upazila), and type of community (host vs refugee) using available population denominators. Population denominators were obtained from annual UNHCR population estimates for the respective catchment areas of the included health facilities and were used to approximate the population at risk. Detailed year-wise catchment population figures, disaggregated by refugee camp and host community union, are provided in S1 Table.

Attack rate ratios (ARR) with corresponding 95% confidence intervals and p-values were calculated to compare scabies burden across sex, age groups, geographic location (Upazila), and community (host vs refugee). P-values were derived by the chi-square test. Proportional morbidity for scabies was calculated as the percentage of all outpatient consultations expressed by month and year.

A multivariable logistic regression analysis was conducted to identify factors associated with infected scabies. Odds ratios (OR) and adjusted odds ratios (AOR) with 95% confidence intervals were estimated. Variables included in the model were sex and age group (0–5 years vs > 5 years).

An Interrupted Time Series (ITS) analysis was performed to assess the impact of MDA on the scabies trend and durability of the impact. The ITS analysis consisted of pre-MDA, MDA phase, and post-MDA segments to estimate changes in level and trend, from January 2021 to December 2024.

We applied a segmented regression approach utilizing ordinary least squares (OLS) estimation to evaluate the changes in both level (immediate effect) and trend (slope over time) associated with the intervention. There were two interruption points: [1] the initiation of the MDA campaign (November 29, 2023), and [2] a second breakpoint at 6 months post-MDA (July 2024), to assess the durability of the intervention effect. The analysis incorporated aggregated weekly counts were used as outcome variable. The independent variables included: a) a continuous time variable representing the underlying pre-MDA trend; b) a binary indicator distinguishing pre- and post-MDA periods to estimate the immediate change in level, c) a continuous variable representing time since MDA initiation to estimate the change in trend during the early post-MDA period, d) a binary indicator for the post–6-month period, and e) a continuous variable representing time since the 6-month breakpoint to estimate the change in trend during the later post-MDA period. The ITS specification can be represented as:


$$\begin{array}{c} Y_{t}=\beta_{\text{0}}+\beta_{\text{1}}Time_{t}+\beta_{\text{2}}PostMDA_{t}+\beta_{\text{3}}TimeAfterMDA_{t}+\beta_{\text{4}}Post\text{6}M_{t}+\beta_{\text{5}}TimeAfter\text{6}M_{t}+\varepsilon_{t} \end{array}$$

where $Y_{t}$ represents the number of scabies cases at week t; $Time_{t}$ denotes the underlying time trend; $PostMDA_{t}$ is a binary indicator for the period following MDA initiation; $TimeAfterMDA_{t}$ represents the number of weeks since MDA initiation; $Post\text{6}M_{t}$ is a binary indicator for the period beyond 6 months post-MDA; and $TimeAfter\text{6}M_{t}$ represents the number of weeks since the 6-month breakpoint.

Regression coefficients were estimated using OLS, and statistical significance was assessed using two-sided p-values. Fitted values and corresponding 95% confidence intervals were derived from the segmented regression and used to visualize observed and estimated trends over time.

Statistical applications, including R (R Foundation for Statistical Computing, Vienna, Austria; version 4.5.1), and Microsoft Excel (Microsoft Corporation, Redmond, WA, USA; version 2602), were used for data analysis and visualization, including tabulation, graphical analyses, and regression analyses.

### Spatial analysis

Geospatial analysis was conducted using QGIS to visualize the distribution of scabies cases and attack rates across refugee camps. Aggregated camp-level case data were linked with administrative boundary shapefiles of Cox’s Bazar camps. Choropleth maps were generated to display attack rates, while proportional symbol maps were used to represent case burden. Spatial patterns and clustering of high-burden camps were assessed through visual interpretation to identify geographic hotspots.

## Results

A total of 384,852 scabies cases were reported from the IOM-supported health facilities in Cox’s Bazar from January 2021 to December 2024. The overall attack rate of scabies over 4 years was 5,562.59 per 10,000, with an average annual attack rate of 1390.65 per 10,000 population (Table 1).

**Table 1:** Distribution of scabies cases and attack rates by demographic characteristics.

Variable	Category	Scabies Count	Percentage (%)	Population	Attack Rate	Attack Rate Ratio	P value
Overall	Total	384,852	100.00	691,857	5,562.59	1.000	–
	Mean age (years)		14.82 ± 16.11 years				
	Median age (years)		9				
Sex	Male	178,917	46.49	338,672	5,282.90	1.000	Ref
	Female	205,935	53.51	353,185	5,830.80	1.104	<0.001
Age group	0 – 5 years	139,747	36.31	124,634	11,212.59	1.000	Ref
	6 – < 18 years	129,406	33.62	232,667	5,561.85	0.496	<0.001
	18 – < 30 years	54,485	14.16	304,642	1,788.49	0.160	<0.001
	30 – 60 years	48,118	12.50	48,002	10,024.17	0.894	<0.001
	60 years+	13,096	3.40	29,914	4,377.88	0.390	<0.001
Upazila	Ukhiya	283,689	73.71	530,560	5,346.97	1.000	Ref
	Teknaf	101,163	26.29	161,297	6,271.85	1.173	<0.001
Community	Host	87,760	22.80	113,659	7,721.34	1.000	Ref
	Refugee	297,092	77.20	578,198	5,138.24	0.665	<0.001

Females accounted for a slightly higher proportion of cases (53.51%) and had a higher attack rate ratio than males (ARR: 1.104, p < 0.001). The highest burden of the cases was reported among children under 5 years (36.31%) and adolescents aged between 6 to <18 years (33.62%). However, the attack rate was significantly higher among the children under the 5-years(11,212.59) in comparison to any other age group and more than twice the overall attack rate. Using this age group as the reference, the attack rate declined significantly with increasing age, 5,561.85 among adolescents (ARR: 0.496, p < 0.001) to 4,377.88 among individuals over 60 years (ARR: 0.390, p < 0.001).

Geographically, about three-quarters of the cases were reported from Ukhiya (73.71%); however, the attack rate was slightly higher in Teknaf (ARR 1.173, p < 0.001). While more than 77% of the cases were reported among the Rohingya refugees, the attack rate was lower than that of the adjacent host communities (ARR 0.665, p < 0.001).

**Fig 1:**
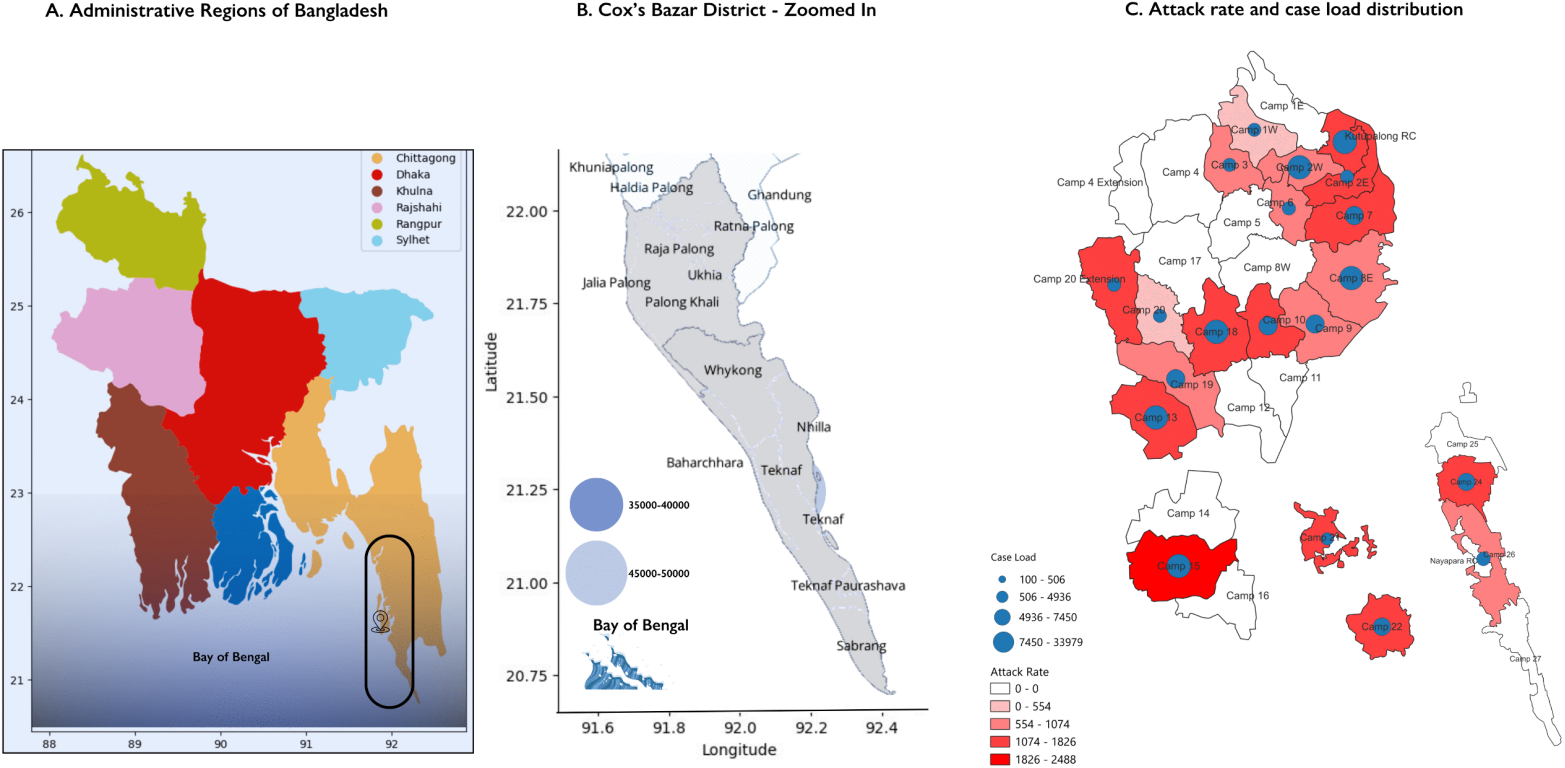
Geospatial distribution of scabies cases and attack rates across catchment camps in Cox’s Bazar, Bangladesh (2021–2024). Panel A: Administrative divisions of Bangladesh showing the location of Cox’s Bazar district. Panel B: Cox’s Bazar district showing Ukhiya and Teknaf upazilas and surrounding unions. Panel C: Camp-level distribution of scabies case load (proportional circles) and attack rate (choropleth shading) across IOM catchment camps. Maps were generated using QGIS (open-source geographic information system; https://qgis.org). Bangladesh administrative boundary and country border shapefiles were obtained from Natural Earth (public domain; https://www.naturalearthdata.com/downloads/10m-cultural-vectors) and the Humanitarian Data Exchange (HDX): https://data.humdata.org/dataset/cod-ab-bgd. Camp boundary shapefiles sourced from the Humanitarian Data Exchange (HDX): https://data.humdata.org/dataset/outline-of-camps-sites-of-rohingya-refugees-in-cox-s-bazar-bangladesh, released under open data licenses compatible with CC BY 4.0. No proprietary basemap services were used.

Fig 1 illustrates the camp-based geo-spatial distribution of scabies cases in the refugee camps of the catchment area. Camps are shaded in red based on total scabies case load with blue circles overlaid based on attack rates (per 100,000). Larger circle size indicates higher attack rate. The highest attack rates were observed in Camp 15 (24,884 per 100,000), Camp 20 Extension (18,259 per 100,000), Kutupalong RC (18,084 per 100,000), and Camp 13 (15,743 per 100,000). In terms of absolute case load, Kutupalong RC reported the highest number of cases (33,979), followed by Camp 15 (13,849) and Camp 13 (8,338).

**Fig 2:**
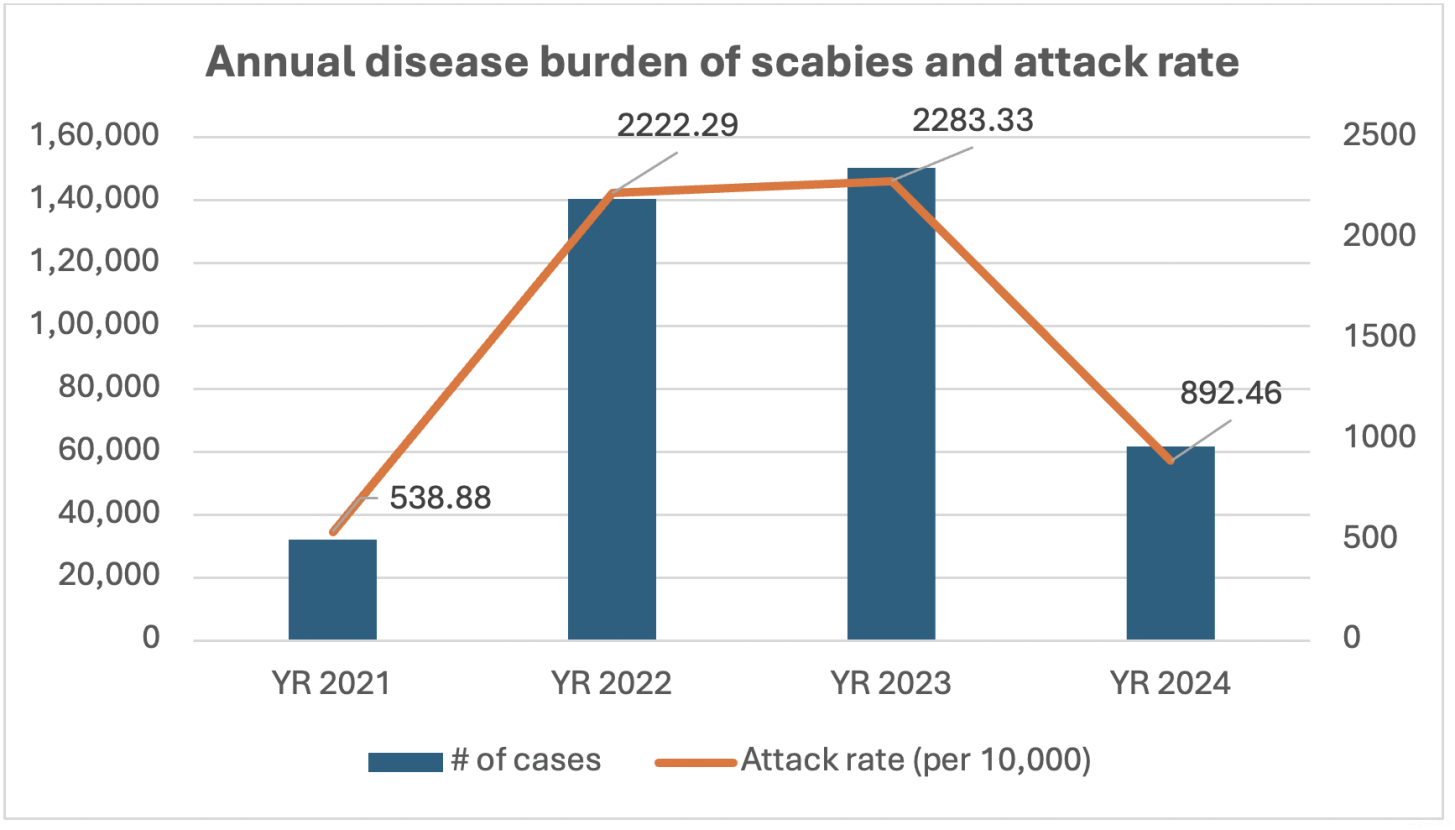
Annual scabies burden and attack rate in Cox’s Bazar, Bangladesh, 2021 to 2024. The catchment population used as the denominator for attack rate calculations was 594,676 in 2021, 632,634 in 2022, 658,993 in 2023, and 691,857 in 2024.

Fig 2 shows that the annual attack rate increased from 538.88 per 10,000 population to a peak of 2283.33 in 2023, which declined to 892.46 in 2024 following the MDA intervention.

**Fig 3:**
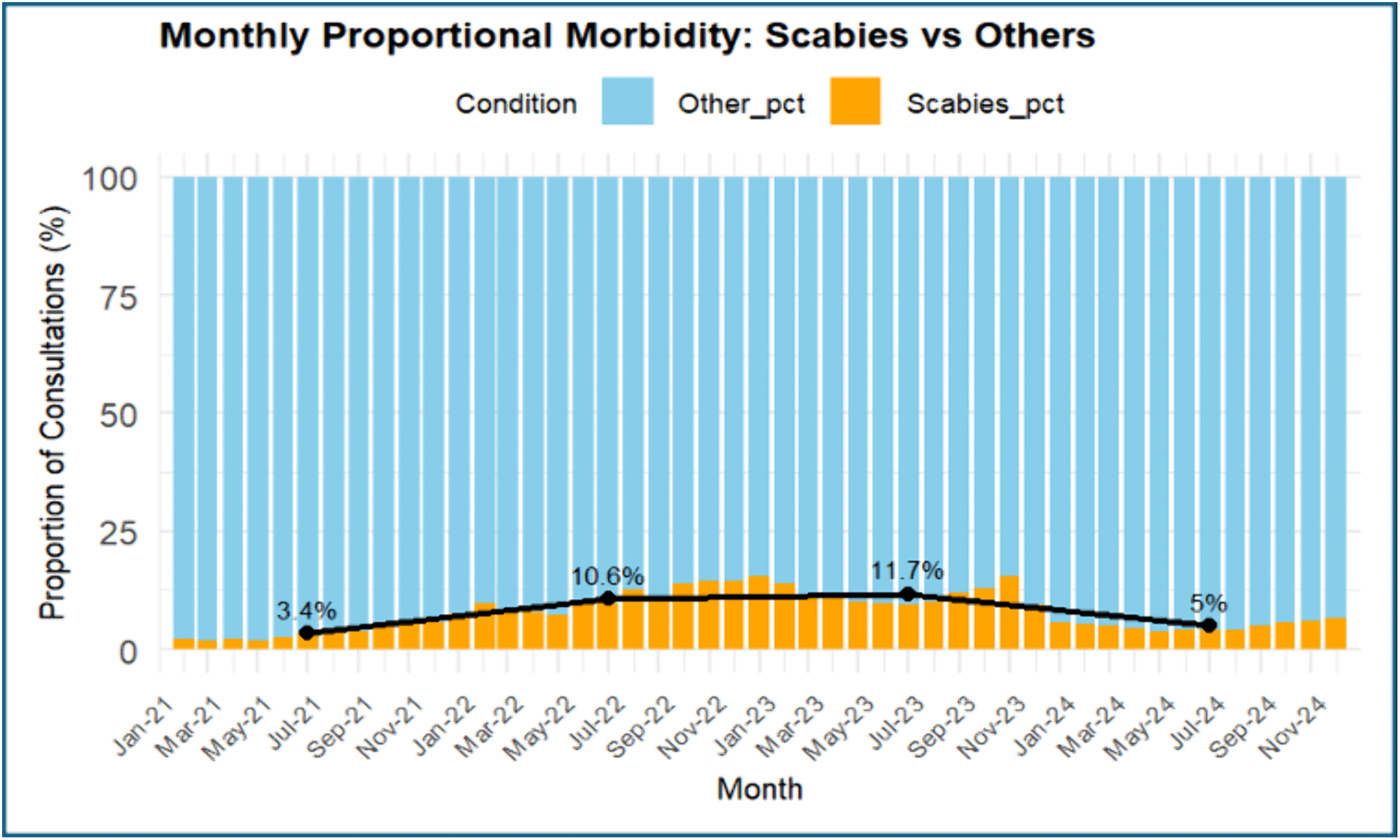
Monthly proportional morbidity (with average morbidity each year): Scabies vs Other Conditions treated at health facilities.

Fig 3 illustrates the monthly proportional morbidity of scabies compared to other reported morbidities from health facilities. The average monthly proportional morbidity of scabies reached a peak of 11.7% in 2023, up from an average of 3.4% in 2021. The average morbidity of scabies dropped to 5% in 2024 following the MDA campaign.

Secondary bacterial infections are a recognized complication of scabies, reported in an estimated 5–20% of cases in high-burden settings [26,27]. In this study, 5.80% of scabies cases were associated with secondary bacterial infections (Table 2). A multivariable regression analysis suggested that male patients had a significantly higher proportion of secondary bacterial infections compared to female patients. The adjusted odds ratio indicated that females had lower odds compared to males (AOR: 1.00 for males vs 0.86 for females, *p* < 0.001). Children under 5 years of age had a higher proportion of secondary bacterial infections compared to other age groups (7.10% vs 5.06%). Individuals aged more than 5 years had significantly lower odds of infected scabies compared to those aged below 5 years (Adjusted OR: 0.68, *p* < 0.001).

**Table 2:** Distribution of scabies by complication (uninfected vs infected scabies) with demographic risk factors.

Variable	Category	Uncomplicated scabies count	Infected scabies Count	Uncomplicated scabies (%)	Infected scabies (%)	OR	Adjusted OR	*p* value
Overall		362,532	22,320	94.20	5.80			
Sex	Male	167,878	11,039	93.83	6.17	1.00 (ref)	1.00 (ref)	<0.001
	Female	194,654	11,281	94.52	5.48	0.86	0.86	
Age Group (0–5 vs > 5)	0–5 years	129,825	9,922	92.90	7.10	1.00 (ref)	1.00 (ref)	<0.001
	>5 years	232,707	12,398	94.94	5.06	0.68	0.68	

**Fig 4:**
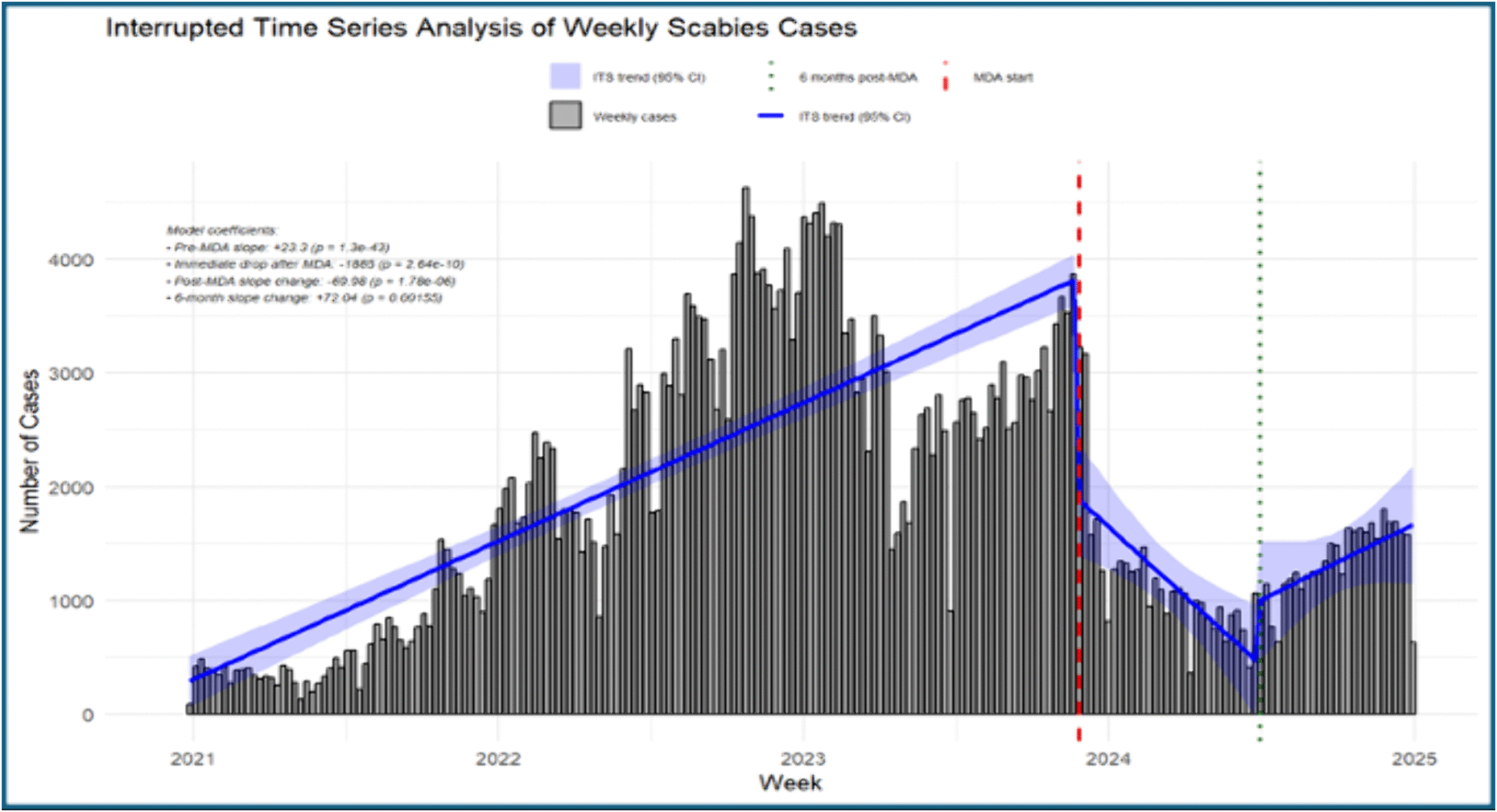
Interrupted Time Series Analysis of Weekly Scabies Trend with pre and post-MDA comparison.

An interrupted time series (ITS) analysis was conducted to evaluate the impact of the MDA campaign on the trend of weekly scabies case count (Fig 4). A significant upward trend was observed from January 2021 until the Mass Drug Administration initiative in December 2023, with an average weekly increase of 23.3 cases (*p* < 0.001). Following the MDA, there was an immediate and sharp decline in level, with a decrease of 1,885 scabies cases (*p* < 0.001). The decline was sustained for 6 months, with a decreasing trend of 69.98 cases per week (*p* < 0.001).

To assess the sustainability of the MDA’s impact, the analysis also included a 6-month post-MDA phase, which revealed a statistically significant upward trend in scabies cases, with an increase of 72.04 cases per week (p = 0.002).

## Discussion

The study found a very high burden of scabies among the Rohingya refugees and adjacent host communities within the catchment areas of the included health facilities in Cox’s Bazar. It was found that the average annual attack rate of scabies was 1390.65 per 10,000 population, equivalent to 13.91%. Household-level surveys in the same setting found the prevalence to range from 39% to as high as 66% [28]. Although the hospital-based attack rate found in the study appears to be lower than that reported in household-level surveys in the same setting, this discrepancy is justifiable because hospital-based data did not capture household contacts/cases not consulted at the health facility level. Scabies, as one of the most prevalent diseases, was also reported in other forced displacement and refugee settings worldwide, including middle east (Syria and Iraq), southern Europe (Italy and Greece), central Europe (Germany) [5]. However, this study reports one of the highest recorded numbers of scabies cases in a single setting.

The study found the highest burden of cases among children and adolescents, with the highest attack rate among children under 5 years. This finding is consistent with a recent study conducted by MSF at its two hospitals in the same setting [8]. A higher susceptibility of children and adolescents to scabies infestation has also been reported in other studies [29,30]. This may be due to that relatively thinner skin of children makes them more vulnerable to scabies mite penetration, there is an increased likelihood of close skin-to-skin contact among children during play or interactions with household members [29,31]. Additionally, children with scabies are more likely to present at health facilities because their symptoms – particularly intense itching and visible lesions — are more distressing and conspicuous than in adults, leading caregivers to seek care. This care-seeking pattern has been observed in similar high-burden settings [8], and may contribute to the higher proportional representation of children in facility-based surveillance data. Such high burden of scabies among children may make them vulnerable to the life-threatening complication of secondary bacterial infections, including acute glomerulonephritis, if untreated [6,19,32,33]. The study also found a higher proportion and attack rate of scabies among women in comparison to men, which also aligns with the findings of the studies in similar settings [34]. This could be attributed to the fact that women, who often accompany their children to the hospital, are overrepresented in the data. The caregiving responsibilities undertaken by women may lead to increased close contact with infected children, thereby heightening their risk of exposure [35]. However, biological susceptibility cannot be excluded and warrants further investigation.

Scabies accounts for a high proportion of morbidity in IOM supported health facilities in the refugee camps, as high as a monthly average of 11.7%. Despite being a treatable condition with topical permethrin and oral ivermectin, the high volume of cases imposes a considerable burden on limited health system resources in this protracted humanitarian context. Given the scale of the burden and ongoing resource constraints, cost-effective and sustainable treatment strategies are essential.

As identified in several studies in the study setting as well as similar refugee contexts worldwide, overcrowded living arrangements and related living conditions are associated with the high disease burden of scabies in the refugee settings [5,7,8,18]. When traditional case management and hygienic measures fail to prevent and control scabies outbreaks, Mass Drug Administration (MDA) is considered one of the crucial public health interventions in such settings to prevent and control scabies outbreaks among large populations. MDA is a promising approach in outbreak settings, preferable over traditional case and contact management, because it treats the entire community simultaneously, thereby interrupting the parasite’s transmission cycle [24,36,37]. Recently published WHO guideline for programme managers on scabies control provides an important operational framework for surveillance, case management, and implementation of MDA strategies, particularly in high-burden and humanitarian settings [38].

As the prevalence rate of scabies in the refugee camps surpassed the crucial 10% WHO threshold necessitating MDA, an MDA campaign was conducted from November 29, 2023, to February 01, 2024 [24]. The effectiveness of MDA in scabies outbreaks has been demonstrated in different settings, including Australia, the Solomon Islands, Fiji, and the Netherlands [19,36,37]. In Ethiopia, a country-wide ivermectin-based MDA was delivered to more than nine million people, representing one of the largest published scabies MDA campaigns globally [39]. Consistent with these findings, this study also observed a substantial reduction of annual attack rate and proportional morbidity, accompanied by a marked decline in weekly case counts following the MDA implementation. Beyond scabies control, ivermectin-based MDA may confer additional collateral benefits, as ivermectin is effective against a range of co-endemic neglected tropical diseases including strongyloidiasis, lymphatic filariasis, and onchocerciasis, potentially amplifying the public health value of a single MDA campaign in co-endemic settings [40].

Further, our analysis also demonstrated that the impact of MDA was sustained in this setting for approximately, six months, after which an increasing trend in cases was observed. This implies that while MDA is effective in rapidly reducing transmission, its impact cannot be sustained over time without improving the broader contextual factors, such as overcrowding, living conditions and hygiene. Therefore, improvements in shelter and living conditions, alongside sustained public health interventions, may help to support the effectiveness of MDA and reduce ongoing transmission of scabies. In addition, Social and Behaviour Change (SBC) interventions may have contributed to treatment uptake and adherence during the campaign. Sustaining such efforts, alongside consideration of periodic MDA cycles at 6–12 monthly intervals, may help to reduce reinfestation and maintain control in this highly dynamic population – particularly given the practical impracticability of addressing structural WASH and housing deficits in the near term [5]. The economic burden of such a large-scale scabies outbreak further reinforces the case for cost-effective preventive strategies: a retrospective costing study from the same setting estimated the total financial cost of IOM’s scabies outbreak response at approximately USD 2.12 million over the outbreak period, with an annual average of USD 531,729 per year, underscoring the considerable resource implications of sustained high caseloads [41].

Additionally, new refugee influxes during or after MDA implementation could have introduced fresh sources of infestation, contributing to the resurgence observed after six months. Another factor that could contribute to the resurgence of scabies is that the MDA campaign covered only refugees, but not the adjacent host community. As revealed in the study, the attack rate was even higher in the surrounding host community than in the refugee community. The possibility of a parallel surge of scabies with possible transmission between camp and host community was also evidenced in other settings [19,27]. Therefore, public health measures should be targeted at both refugees and host communities to halt the transmission of the disease.

## Limitations of the study

The study only included data from IOM-supported health facilities in the refugee camps. Although, as the denominator, we have only taken into account the catchment population of these facilities, the prevalence and attack rate in this study could be underrepresented, as the study could not capture data from facilities run by other partners in the same catchment. While IOM facilities are strategically located throughout the refugee camps, our study only covered areas within their catchment zones and may not be representative of all refugee camps or the host community.

Population denominators were derived from annual UNHCR estimates for the catchment areas of the included health facilities. Given the dynamic nature of the camp population, including movement between camps and small scale influxes, these estimates may not fully capture short-term population fluctuations. As a result, the calculated attack rates may be subject to over- or under-estimation and should be interpreted as approximate measures of disease burden rather than precise population-based incidence rates.

Additionally, due to the retrospective nature of the study, we were unable to capture data on some relevant variables, such as behavioral patterns, household contacts, and cases. Despite some limitations, the study provided valuable insights into the epidemiology and magnitude of scabies, as well as the impact of MDA on the outbreak. The study did not attempt to predict the optimal frequency of future MDA rounds, which is critical given the observed resurgence. Further modeling studies are needed to determine appropriate MDA intervals under current camp conditions.

## Conclusion

The study revealed a high burden of scabies among the Rohingya refugees and the adjacent host community, requiring urgent public health attention. The study found higher vulnerability among children and women, which will necessitate targeted interventions. Furthermore, the study found Mass Drug Administration to be an effective approach for a rapid and substantial reduction in the disease burden. However, the resurgence of the upward trend in scabies indicates that the impact of MDA cannot be sustained if the underlying factors of scabies outbreaks, including overcrowding, water, sanitation, and hygiene, are not addressed. Therefore, we urge policymakers and humanitarian actors to adopt a holistic and coordinated approach to halt the transmission of this disease.

## Supporting information

S1 Table Year-wise catchment population figures for IOM-supported health facilities in Ukhiya and Teknaf, Cox’s Bazar (2021–2024). (XLSX)

S1 Data Cleaned anonymized scabies case dataset used for the interrupted time series and descriptive analyses, Cox’s Bazar refugee camps, 2021–2024. The dataset includes anonymized, aggregated scabies case counts by demographic characteristics, weekly and monthly totals, infection status, and variables used in the analyses.
(XLSX)
