# Supplementary material for: Massive scabies outbreak in Rohingya refugee camps, Cox’s Bazar: Epidemiology and impact of a mass drug administration (MDA) campaign – A retrospective study
Source: PLoS Negl Trop Dis. 2026 Jul 1;20(7):e0013832. doi: 10.1371/journal.pntd.0013832 (PMC13349296; doi:10.1371/journal.pntd.0013832)
Supplement: Attachment — Submitted filename: Response to reviewers.docx [file pntd.0013832.s005.docx]

**Response to Reviewers**

**Manuscript ID: PNTD-D-25-02182R1**

**Manuscript Title: Massive scabies outbreak in Rohingya refugee camps, Cox's Bazar: Epidemiology and impact of a mass drug administration (MDA) campaign – a retrospective study**

We sincerely appreciate the efforts from the editor and peer reviewers for the valuable comments and constructive feedback. Below are the line-by-line responses to the comments.

**Responses to Reviewers**

**Reviewer #1:**

**Comment 1: “The manuscript is improved, and suitable for publication.”**

Response: We sincerely thank the reviewer for the positive evaluation and encouraging comments on our manuscript.

**Reviewer #2**

**Comment 1: “It might be possible to reduce the number of figures.”**

**Response:** Thank you for the suggestion. We carefully reviewed the figures. However, we suggest to retain the current number of figures as each figure presents distinct characteristics: a) geographical distribution, b) yearly trend, c) proportional morbidity, and d) ITS – impact of MDA. Therefore, we request the peer reviewer and the editor to allow us to keep the current number of figures (four) for comprehensive interpretation of the epidemiology and MDA impact.

**Comment 2: “I think it would be appropriate to include reference to the largest MDA published so far: Enbiale W, Baynie TB, Ayalew A… ‘Stopping the itch’: mass drug administration for scabies outbreak control covered for over nine million people in Ethiopia…”**

Response: Thank you for this valuable suggestion. We have now incorporated the recommended Ethiopian MDA study into the discussion section. Reference # 39.

**Comment 3: Comment: “and to sign post the recently launched WHO ‘Control of Scabies; A guide for national program managers’.”**

**Response:** Thanks for the comment. We have now referenced the recently launched WHO “Control of Scabies: A Guide for National Program Managers” in the Discussion section. Reference # 38.

**Comment 4: A useful review of the management of the massive outbreak in CB by the local teams. Given the very hot off the press WHO guide for program managers for Control of Scabies, you could sign post to this in your discussion/conclusion as this now provides useful direction that you may well have appreciated at the time of your interventions. Also noting your large number of cases treated, aware that the Ethiopian experience which whilst was not in a displaced camp setting, was non the less impressive in its reach, I suggest referencing this (see above)**

Response: Thank you for the positive feedback. Comments on referencing WHO guide, and Ethiopian study are addressed above.

We hope that our revisions have adequately addressed the comments.
