# Supplementary material for: Massive scabies outbreak in Rohingya refugee camps, Cox’s Bazar: Epidemiology and impact of a mass drug administration (MDA) campaign – A retrospective study
Source: PLoS Negl Trop Dis. 2026 Jul 1;20(7):e0013832. doi: 10.1371/journal.pntd.0013832 (PMC13349296; doi:10.1371/journal.pntd.0013832)
Supplement: Attachment — Submitted filename: Response_to_Reviewers_PNTD-D-25-02182_v3.docx [file pntd.0013832.s004.docx]

March 26, 2026

**PLOS Neglected Tropical Diseases**

Manuscript ID: PNTD-D-25-02182

Title: Massive Scabies Outbreak in Rohingya Refugee Camps, Cox's Bazar: Epidemiology and Impact of a Mass Drug Administration (MDA) Campaign — A Retrospective Study

**Response to Reviewers**

Dear Dr. Michael Marks, Dr. Adly Abd-Alla, and the Editorial Team of PLOS Neglected Tropical Diseases,

We sincerely thank the Academic Editor, Section Editor, and all three reviewers for their thorough and constructive assessment of our manuscript. The comments have been invaluable in strengthening the scientific quality, clarity, and rigour of the work. We have carefully addressed every point raised and revised the manuscript accordingly. All changes are highlighted in the revised manuscript with track changes enabled.

Below, we provide a point-by-point response to every comment. Reviewer comments are shown in **red italic**, our responses in **blue**, and specific text changes made in the manuscript in **green italic**.

**ADDITIONAL EDITOR COMMENTS**

***Editor Comment 1: Writing quality***

**Reviewer comment:** *The writing throughout would benefit from re-reading.*

**Response:** We have carefully re-read the entire manuscript and revised the language for clarity, consistency, and precision. Awkward constructions, redundant phrases, and typographical errors have been corrected. Tense consistency has been enforced throughout, particularly in the Results and Conclusion sections.

***Editor Comment 2: Water/WASH and scabies risk***

**Reviewer comment:** *There is little evidence to support the suggestion (line 65) that access to water is important for scabies. Washing does not remove scabies mites. Overcrowding is a risk factor but the evidence linking this directly to water access is very poor.*

**Response:** We thank the editor for this important scientific correction. We have revised the relevant sentence in the Introduction to remove any implication that WASH directly reduces scabies transmission. The revised text focuses on overcrowded living conditions and associated constraints as primary risk factors, with WASH framed in the context of broader living conditions rather than direct scabies transmission.

**Revised text:** *"...refugees and displaced communities face an elevated risk of transmission due to overcrowded living conditions that facilitate close skin-to-skin contact, as well as broader constraints in living conditions, challenges in maintaining personal hygiene and laundering clothes and bedding, limited access to healthcare, shortage of scabicidal drugs, and socio-cultural factors such as language barriers and stigma (5,7–9)."*

***Editor Comment 3: MDA inadequately described***

**Reviewer comment:** *The MDA is inadequately described. There is no discussion of how the denominator population was ascertained, how the MDA was actually conducted, the coverage achieved, the time frame over which it was conducted.*

**Response:** The MDA section in the Methods has been substantially expanded to include: (1) the denominator population derived from UNHCR camp-level estimates; (2) the MDA regimen (two doses oral ivermectin 200 µg/kg, 7–14 days apart, with 5% permethrin for contraindicated individuals); (3) the DOT delivery strategy; (4) workforce deployment (100 healthcare workers, 130 supervisors, >1,600 community health workers); (5) the MDA Advisory Committee structure; (6) drug quantities distributed; and (7) coverage exceeding 99% of target for both doses, referenced by citations 24 and 25.

***Editor Comment 4: ITS statistical methodology***

**Reviewer comment:** *The statistical methodology of the ITS is not adequately described. An ITS can look for an immediate effect but also an alteration of the overall trend — it is unclear what was looked for, how the ITS was fitted.*

**Response:** The ITS methodology has been substantially expanded. The revised text now clearly describes: (1) the segmented regression approach using OLS estimation; (2) assessment of both level changes (immediate effect) and trend changes (slope); (3) the two interruption points used (MDA initiation and 6-month post-MDA breakpoint); (4) all independent variables in the model; and (5) the full regression equation with notation for each term. The analysis captures the immediate drop in level and changes in slope across three periods: pre-MDA, early post-MDA, and late post-MDA.

***Editor Comment 5: Statistical software versions***

**Reviewer comment:** *The description of the statistical software is lacking — version numbers should be provided. NB R Studio is also just an interface, the analysis software is R.*

**Response:** Updated. The revised text reads: "Statistical applications, including R (R Foundation for Statistical Computing, Vienna, Austria; version 4.5.1), and Microsoft Excel (Microsoft Corporation, Redmond, WA, USA; version 2602), were used for data analysis and visualization." RStudio is correctly noted as an interface only.

***Editor Comment 6: Ethics — WHO and IOM involvement***

**Reviewer comment:** *Was the only ethics from a local hospital? Presumably WHO and IOM have ethics committees who were involved in the design and evaluation of the MDA intervention.*

**Response:** The Ethics section has been substantially revised to clarify the full governance structure: (1) local ethics clearance from Cox's Bazar Medical College Hospital (CoxMC/2023/017); (2) coordination with and clearance from the Office of the Civil Surgeon and the RRRC Health Coordinator; (3) formal approval for the MDA campaign from the Government of Bangladesh and technical clearance from WHO Country Office and WHO SEARO; and (4) internal programmatic authorization from the IOM Migration Health Division Research Unit for both the study and retrospective programme data use.

***Editor Comment 7: Population movement in calculating case rates***

**Reviewer comment:** *How do you account for population movement in calculating your case rates? It seems like you have taken a fixed population as the denominator but included all cases. This is going to over-estimate the number of cases per population.*

**Response:** We acknowledge this important methodological limitation. Annual UNHCR population estimates were used as denominators for each year, which do not capture within-year population movements, inter-camp movements, or small-scale influxes. A dedicated paragraph in the Limitations section explicitly addresses this, noting that the use of fixed annual denominators may result in over- or under-estimation of attack rates, and that results should be interpreted as approximate measures of disease burden rather than precise population-based incidence rates.

***Editor Comment 8: Geospatial analysis not in Methods***

**Reviewer comment:** *In the results you present a geospatial analysis which was never described in the methods.*

**Response:** A dedicated Spatial Analysis subsection has been added to the Methods, describing: use of QGIS for geospatial visualization; linking of aggregated camp-level case data with administrative boundary shapefiles; choropleth maps for attack rates; proportional symbol maps for case burden; and visual interpretation for hotspot identification.

***Editor Comment 9: Other analyses not mentioned in Methods***

**Reviewer comment:** *Similarly there are several other analyses that are never mentioned in the methods (for example bacterial infections).*

**Response:** The Statistical Analysis section has been updated to ensure all analyses reported in the Results are described in the Methods. The logistic regression analysis for secondary bacterial infections (with OR and AOR estimates, variables including sex and age group) and proportional morbidity calculation are now explicitly described in the Methods.

**REVIEWER 1 COMMENTS**

Reviewer 1 was broadly positive, noting the study addresses a highly relevant topic with clear design, structure, and conclusions. Only minor drawbacks were raised.

***Reviewer 1, Comment 1: Use of "intricately"***

**Reviewer comment:** *In the introduction, I don't think it's necessary nor accurate to use the adjective "intricately" in: "which intricately burrow..."*

**Response:** Agreed. The word has been removed. The revised text reads: "It is a parasitic skin disease caused by the Sarcoptes scabiei mite, which burrows into the skin, lays eggs, and incites severe pruritus."

***Reviewer 1, Comment 2: Ivermectin dosing sentence***

**Reviewer comment:** *The sentence may say WHO recommends two annual rounds of IVM, which does not correspond to reality.*

**Response:** Corrected. The revised text reads: "The MDA regimen consisted of two doses of oral ivermectin at 200 µg/kg body weight, with an interval of 7–14 days between doses."

***Reviewer 1, Comment 3: "Responsible" and reference accuracy***

**Reviewer comment:** *"Responsible" should be replaced with "associated factors". Also: is reference 8 appropriate?*

**Response:** "Responsible" has been replaced with "associated with" throughout. References for this sentence have been reviewed and updated to ensure they specifically support the claim about overcrowded living conditions in refugee settings.

***Reviewer 1, Comment 4: Case-by-case management***

**Reviewer comment:** *The traditional scabies approach is not based on case-by-case management, but rather on case and contact management.*

**Response:** Corrected. Revised text: "MDA is a promising approach in outbreak settings, preferable over traditional case and contact management, because it treats the entire community simultaneously, thereby interrupting the parasite's transmission cycle."

**REVIEWER 2 COMMENTS (Dr. Lucinda Claire Fuller)**

***Reviewer 2: Line 88 — typo***

**Reviewer comment:** *Line 88 — small typo: take out "to)"*

**Response:** Corrected.

***Reviewer 2: Line 127 — MSF abbreviation***

**Reviewer comment:** *Line 127: "MSF" — not previously explained.*

**Response:** Corrected. Full name "Médecins Sans Frontières (MSF)" is now used at first mention.

***Reviewer 2: Why permethrin mainly used***

**Reviewer comment:** *Line 128 — do you know why permethrin was mainly used?*

**Response:** Permethrin 5% topical cream was the primary treatment as it is the recommended first-line agent for uncomplicated scabies, particularly suitable for children and pregnant women in whom oral ivermectin is contraindicated. It was also the predominant medication in IOM facility stockpiles during the study period. Oral ivermectin and benzyl benzoate were used selectively based on clinical indication and availability. This is reflected in the Methods text.

***Reviewer 2: MDA coverage and broken reference link***

**Reviewer comment:** *Do you know the % reach of the MDA? Please check the reference link — I don't think it works anymore.*

**Response:** Coverage exceeding 99% of target for both doses has been added, supported by references 24 (WHO Bangladesh MDA news article) and 25 (Health Sector Cox's Bazar Bulletin, March 2024). All reference links have been verified and access dates updated to March 2026.

***Reviewer 2: Line 186 — sentence check***

**Reviewer comment:** *Line 186: sentence check — may need to insert "that"*

**Response:** The sentence has been reviewed and corrected grammatically.

***Reviewer 2: Table 2 — bar chart suggestion***

**Reviewer comment:** *Table 2: I think it would be more eye-catching as a bar chart.*

**Response:** We appreciate this suggestion. Table 2 has been retained in tabular format to ensure precise numerical reporting of all variables (counts, percentages, OR, AOR, p-values) as required for reproducibility. We note this as a potential enhancement for future publications.

***Reviewer 2: Table 3 — variable labelling***

**Reviewer comment:** *Table 3: suggest variables are either "infected and not infected" or "uncomplicated and infected"*

**Response:** Column headers in Table 2 have been standardised to use "Uncomplicated scabies" and "Infected scabies" consistently throughout the table title, headers, and in-text references.

***Reviewer 2: Greece data repetition***

**Reviewer comment:** *Repetition of the Greece data sentence line 69 and 238 — suggest removing.*

**Response:** The repeated Greece comparison sentence has been removed from the Discussion. The Introduction retains the original Greece reference for context.

***Reviewer 2: Line 244 — children symptomatic and care-seeking***

**Reviewer comment:** *Could it be that more children are symptomatic and prone to secondary infection which is why they are brought to the clinics — similar to indigenous Australian data.*

**Response:** This important insight has been added to the Discussion: "Additionally, children with scabies are more likely to present at health facilities because their symptoms — particularly intense itching and visible lesions — are more distressing and conspicuous than in adults, leading caregivers to seek care. This care-seeking pattern has been observed in similar high-burden settings (8), and may contribute to the higher proportional representation of children in facility-based surveillance data."

***Reviewer 2: Line 271 — ivermectin NTD benefits***

**Reviewer comment:** *Do you want to consider including the additional benefits of ivermectin being effective against a range of NTDs?*

**Response:** Added to Discussion: "Beyond scabies control, ivermectin-based MDA may confer additional collateral benefits, as ivermectin is effective against a range of co-endemic neglected tropical diseases including strongyloidiasis, lymphatic filariasis, and onchocerciasis, potentially amplifying the public health value of a single MDA campaign in co-endemic settings (38)." Reference: Crump & Omura, 2011.

***Reviewer 2: Line 280 — MDA cycles and WASH impracticability***

**Reviewer comment:** *You advise addressing social determinants of health but could also recommend regular cycles of MDA at 6–12 monthly to address incoming cases, and/or the impracticalities of addressing WASH etc.*

**Response:** Revised to explicitly recommend regular MDA cycles at 6–12 monthly intervals and acknowledge structural WASH and housing constraints. The economic burden sentence with USD 2.12 million total cost has also been added, citing the companion costing study (Ref 39).

***Reviewer 2: References — broken links***

**Reviewer comment:** *References in general: please check the links as July 2025 is sometime ago and the one I checked didn't work.*

**Response:** All reference URLs have been verified. Non-functional links have been updated. Access dates for all online references have been updated from July 2025 to March 2026.

***Reviewer 2: WHO Programme Managers Guide***

**Reviewer comment:** *I know we are awaiting the WHO Programme Managers Guide to scabies outbreaks — it should be available soon and would be a great reference.*

**Response:** As of March 2026, this document has not yet been formally published. We have added the following sentence to the Discussion: "Formal WHO programme-level guidance on scabies control is anticipated and, once published, will further support evidence-based planning for MDA deployment in humanitarian settings." We will update this reference if it becomes available before acceptance.

**REVIEWER 3 COMMENTS — LINE BY LINE**

Reviewer 3 provided extensive line-by-line comments across all sections. We have addressed each point below in the order raised.

***R3 — General Comments***

**Reviewer comment:** *The writeup contains a number of statements that are poorly referenced or not referenced at all. Authors should consider rewriting especially introduction and discussion portions. Authors should opt for simpler clearer language. The outcome exposes the crucial need for incorporation of behavioural, attitudinal and socioeconomic factors.*

**Response:** We have carefully revised the Introduction and Discussion for clarity, language, and referencing. Every factual claim now has an appropriate citation. We acknowledge the importance of behavioural and socioeconomic factors in scabies transmission, and this context has been strengthened in the Discussion, particularly in relation to overcrowding, hygiene constraints, and social factors. We note that this retrospective study was limited to routine clinical surveillance data and did not prospectively collect behavioural or socioeconomic variables; this limitation is now explicitly stated.

***R3 — Abstract, Background: responsible body for MDA***

**Reviewer comment:** *It may be important to state here the body responsible for the MDA campaign (i.e., authors? MoH?)*

**Response:** Revised. The Abstract Background now reads: "In response to the massive outbreak, an extensive Mass Drug Administration (MDA) campaign was conducted by WHO Bangladesh, in collaboration with the Government of Bangladesh and Health Sector partners, from November 29, 2023, to February 01, 2024."

***R3 — Abstract, Objectives: rewrite sentence 1 (line 29)***

**Reviewer comment:** *Consider rewriting sentence 1 (line 29): "The objectives of the study were: a) to determine the epidemiological characteristics (i.e., magnitude, age-sex distribution and attack rate) of the scabies outbreak..."*

**Response:** Revised. The Objectives in the Abstract now read: "The objectives of the study were: a) to determine the epidemiological characteristics (i.e., magnitude, age-sex distribution, and attack rate) of the scabies outbreak in the Rohingya refugee camps; and b) to evaluate the impact and durability of MDA in reducing the burden of scabies in the refugee camps."

***R3 — Abstract, Methodology: more detail on IOM data and MDA type***

**Reviewer comment:** *What exact information/details is included in the IOM data that was used? MDA is mentioned at the end of results but no detail is provided on the type of MDA.*

**Response:** The Abstract Methodology now specifies the data variables (age, sex, camp location, secondary infection status) and identifies the MDA as ivermectin-based with evaluation using interrupted time series analysis.

***R3 — Abstract, Results: % decline (lines 40–41 and 43)***

**Reviewer comment:** *Lines 40–41 and 43: % decrease could be presented to distinctively show the rate of decline of attacks.*

**Response:** Revised to include percentage framing: "...representing a reduction of 1,885 cases per week (p < 0.001) — equivalent to a weekly decline of approximately 100% from the pre-MDA peak."

***R3 — Abstract, Conclusion: line 49***

**Reviewer comment:** *Line 49: ...was an effective approach for a rapid and...*

**Response:** Corrected. The Conclusion now reads: "MDA was an effective approach for a rapid and substantial reduction of the disease burden."

***R3 — Introduction, line 57: referencing style and WHO***

**Reviewer comment:** *Line 57: check referencing style. Delete (WHO) or replace with the clear sentence "according to the World Health Organization"*

**Response:** Revised. The sentence now reads: "Scabies, classified among neglected tropical diseases, impacts over 400 million individuals annually (1,2)." The parenthetical (WHO) has been removed; the citation handles the attribution.

***R3 — Introduction, line 57: Sarcoptes scabiei italicisation***

**Reviewer comment:** *Line 57: Sarcoptes scabiei — italicize as it is a scientific name.*

**Response:** Corrected. Sarcoptes scabiei is now italicised at all occurrences throughout the manuscript.

***R3 — Introduction, line 59: literature for skin-to-skin contact***

**Reviewer comment:** *Line 59: Provide relevant literature to support documented mode of transmission i.e. skin-to-skin contact.*

**Response:** References 3 (Murray & Crane, StatPearls) and 4 (IACS, About Scabies) now support the skin-to-skin contact transmission statement.

***R3 — Introduction, line 63: "confront"***

**Reviewer comment:** *Line 63: The word "confront" is poorly used in this context. Revise.*

**Response:** Replaced with "face". Revised text: "...which significantly increase vulnerability to infectious disease outbreaks that the camp populations face."

***R3 — Introduction, line 67: context/background before introducing Rohingya***

**Reviewer comment:** *Prior to the introduction of Rohingya into the conversation (in line 67), it would be better to provide some context/introduction/background of the camp.*

**Response:** We have restructured the Introduction paragraph so that a brief contextual description of the Cox's Bazar setting and camp conditions precedes the specific Rohingya population data. The text now establishes the humanitarian context before introducing the population figures.

***R3 — Introduction, lines 66–67: overly stretched context and references***

**Reviewer comment:** *Lines 66–67: The context provided is overly stretched. It is better written as: "Such prevalence of scabies is seen in the Rohingya refugee camps in Bangladesh and this could be attributed to..." Provide relevant proof/reference for claims.*

**Response:** The introduction paragraph has been restructured for conciseness. The scabies burden in the camps is now introduced more directly, with references supporting each factual claim.

***R3 — Introduction, lines 68–69: consistency in prevalence presentation***

**Reviewer comment:** *Lines 68–69: Ensure consistency in presentation of historical prevalence rates. Stick to percentages or cases per year.*

**Response:** Revised to ensure consistency. All prevalence comparisons in the Introduction are now presented as percentages for uniformity.

***R3 — Introduction, lines 70–75: structural reordering***

**Reviewer comment:** *Lines 70–75 could be moved to just before line 66 for better reading and context. The introduction looks very jointed and reads poorly.*

**Response:** We have restructured the Introduction to improve logical flow: the camp background context now precedes the description of scabies burden, and the transition from global scabies burden to the Rohingya setting is smoother.

***R3 — Introduction, lines 75–77: repetition***

**Reviewer comment:** *Lines 75–76 looks out of place. Lines 76–77: the sentences are beginning to repeat — this assertion is already made in lines 66–67.*

**Response:** The repeated assertion has been removed. The Introduction no longer repeats the statement about high demand for medical attention and financial strain that was already made earlier in the paragraph.

***R3 — Introduction, lines 82–83: delete "globally" and rewrite***

**Reviewer comment:** *Lines 82–83, the word "globally" must be deleted. Also, consider rewriting for clarity.*

**Response:** "Globally" has been deleted. The sentence has been rewritten for clarity.

***R3 — Introduction, lines 82–86: move to after line 94***

**Reviewer comment:** *Move the content of lines 82–86 to after line 94. The former shows the study's problem statement and justification and links better with line 95.*

**Response:** The problem statement and study justification paragraph has been repositioned to follow the description of the MDA campaign, creating a more logical progression from the problem (scabies outbreak) → response (MDA) → gap in knowledge → study rationale.

***R3 — Introduction, line 89: state the country***

**Reviewer comment:** *Line 89: state the country of the camps.*

**Response:** The country of the camps (Bangladesh) is now explicitly stated: "...33 camps in Cox's Bazar and Bhasan Char Island in Bangladesh."

***R3 — Introduction, line 100: delete "In summary"***

**Reviewer comment:** *Line 100: Delete the phrase "In summary". Be succinct.*

**Response:** "In summary" has been deleted.

***R3 — Introduction, line 106: indicate jurisdiction***

**Reviewer comment:** *Line 106: In which jurisdiction(s) will these findings be most useful. Indicate.*

**Response:** The objectives paragraph now concludes: "The findings of the research will assist health programmes and policymakers in predicting the scale and characteristics of scabies outbreaks...thereby facilitating proper planning for the effective integration of scabies prevention and management within the essential health service model in Bangladesh and similar humanitarian and refugee settings globally."

***R3 — Methods: clinical diagnosis criteria***

**Reviewer comment:** *The criteria for clinical diagnosis of scabies and secondary bacterial infections should be clearly stated.*

**Response:** A detailed paragraph has been added defining clinical diagnostic criteria for scabies (pruritus worse at night, papules/burrows/vesicles in typical distribution sites, household contact evidence, per MSF guidelines and IACS 2020 criteria) and secondary bacterial infection (pustules, crusting, oozing, impetiginization at lesion sites during routine clinical encounter).

***R3 — Methods: MDA threshold, planning, and delivery***

**Reviewer comment:** *Authors need to indicate clearly the point at which the threshold for MDA was met/what guided the decision. Further details needed on planning, engagement, targeted population, and delivery personnel.*

**Response:** The MDA section has been comprehensively expanded: (1) WHO May 2023 prevalence survey reporting 39%, exceeding the 10% WHO threshold; (2) targeted population of approximately 992,500 across 33 camps; (3) DOT delivery strategy; (4) pre-implementation training of 100 healthcare workers, 130 supervisors, and over 1,600 community health workers; (5) MDA Implementation Advisory Committee structure; and (6) 68 storage locations including 48 health facilities.

***R3 — Methods, line 114: "at any/either one of the 35"***

**Reviewer comment:** *Line 114: ...at any/either one of the 35...*

**Response:** Corrected. Text now reads: "...received care at any of the 35 IOM-supported health facilities..."

***R3 — Methods, lines 115–117: delete or move to Introduction***

**Reviewer comment:** *Lines 115–117: Delete or use to supplement content in introduction.*

**Response:** These lines have been reviewed and any content that duplicated the Introduction has been removed from the Methods. Contextual information on the camp setting now appears appropriately in the Introduction only.

***R3 — Methods, line 122: "detection" or "diagnosis" instead of "presence"***

**Reviewer comment:** *Line 122: Presence of secondary infection? Doesn't "detection" or "diagnosis" read better?*

**Response:** Revised. "Infected scabies cases were defined as scabies cases presenting with clinical signs consistent with a diagnosis of secondary bacterial infection."

***R3 — Methods, line 123: "centrally cleaned"***

**Reviewer comment:** *Line 123: What does "centrally cleaned" mean? Simplicity is better.*

**Response:** Revised to: "The data were verified and cleaned daily at a central level before being exported to an Excel spreadsheet for analysis."

***R3 — Methods, lines 125–128: clinical diagnosis definitions***

**Reviewer comment:** *Lines 125–128: Authors should state clearly what definitions were used for the clinical diagnosis of scabies.*

**Response:** A full paragraph has been added defining clinical diagnostic criteria — addressed above under the Methods clinical diagnosis point.

***R3 — Methods, line 139: "Therapeutics"***

**Reviewer comment:** *Line 139: "Therapeutics" is poorly used here. Authors should stick to e.g. "medication".*

**Response:** "Therapeutics" has been replaced with "medications" throughout.

***R3 — Methods, lines 138–142: logistics vs MDA conduct***

**Reviewer comment:** *Lines 138–142 read more like logistics than a report of the reach and mode of conduct of the MDA.*

**Response:** The MDA section has been restructured. Logistical details (drug quantities, storage locations) are presented concisely, while the primary description focuses on the reach (992,500 individuals), coverage (>99%), and mode of delivery (DOT strategy, trained workforce).

***R3 — Methods, line 146: catchment populations in methods***

**Reviewer comment:** *Line 146: If respective catchment populations are to be used, they must be highlighted somewhere in the methods section.*

**Response:** Added to Statistical Analysis section: "Detailed year-wise and month-wise catchment population figures, including both refugee and host community populations, are provided in Supplementary Table S1."

***R3 — Methods, line 157: software versions***

**Reviewer comment:** *Line 157: Kindly report the versions and manufacturer(s)/origins of the statistical tools used.*

**Response:** Full software details now included: "R (R Foundation for Statistical Computing, Vienna, Austria; version 4.5.1)" and "Microsoft Excel (Microsoft Corporation, Redmond, WA, USA; version 2602)".

***R3 — Results, Table 1: formatting***

**Reviewer comment:** *Table 1: Format text spaces in the table and entire table for easier reading and understanding.*

**Response:** Table 1 has been reformatted for improved readability. Column widths, text alignment, and spacing have been adjusted. Merged cells and consistent formatting have been applied throughout.

***R3 — Results, line 186: sentence structure***

**Reviewer comment:** *Line 186: ...which was performed...*

**Response:** The sentence has been corrected to read: "An interrupted time series (ITS) analysis was conducted to evaluate the impact of the MDA campaign on the trend of weekly scabies case count."

***R3 — Results, tense and syntax throughout***

**Reviewer comment:** *Throughout section, consider the tense/syntax of the sentences.*

**Response:** All present-tense verbs in the Results section that should be past tense have been corrected for consistency with standard scientific reporting.

***R3 — Results, method of data presentation***

**Reviewer comment:** *Method of presentation of data in the context/section is poor. Provide clear, easily quantifiable figures. Finer details can then be in methodology section.*

**Response:** The Results section has been revised to lead with clear quantitative findings. Supporting methodological details that were embedded in the Results have been moved to or cross-referenced with the Methods section.

***R3 — Results, lines 203–204***

**Reviewer comment:** *Lines 203–204 not necessary as there is a section for reporting that outcome.*

**Response:** The redundant sentence has been removed. A contextual statement has been added immediately before Table 2: "Secondary bacterial infections are a recognized complication of scabies, reported in an estimated 5–20% of cases in high-burden settings (26,27). In this study, 5.80% of scabies cases were associated with secondary bacterial infections (Table 2)."

***R3 — Results: secondary infection context in Introduction***

**Reviewer comment:** *While presentation of the secondary bacterial infection section is important, context must be provided in the introduction section.*

**Response:** The Introduction now includes a strengthened sentence about secondary bacterial infections as a significant complication of scabies, with reference to immune-mediated sequelae including acute post-streptococcal glomerulonephritis, rheumatic heart disease, and death (References 2, 3, 5, 6).

***R3 — Results, Table 3 (Table 2 in current manuscript)***

**Reviewer comment:** *Table 3: same issues as Table 1 and Table 2.*

**Response:** Table 2 (previously Table 3) has been reformatted consistently with Table 1 — adjusted column widths, text alignment, and uniform cell formatting applied.

***R3 — Results, Figure 1: not in Methods***

**Reviewer comment:** *Figure 1: this approach is not mentioned in the methods/analysis.*

**Response:** A dedicated Spatial Analysis subsection has been added to the Methods, fully describing the QGIS-based geospatial analysis approach.

***R3 — Discussion, line 231: "adjacent host communities"***

**Reviewer comment:** *Line 231: The use of the word "and adjacent host communities" suggests data was collected from these areas as well.*

**Response:** Revised for clarity. The Discussion now explicitly states: "The study found a very high burden of scabies among the Rohingya refugees and the adjacent host communities within the catchment areas of the included health facilities." A clarifying sentence in the Limitations notes that host community data were captured only through IOM health facilities serving those communities, which are located adjacent to the refugee camps.

***R3 — Discussion, line 237: NTDs investigation***

**Reviewer comment:** *Line 237: Was there an investigation on a number of NTDs or their prevalence listed in the results? If not, consider removing.*

**Response:** The sentence in question has been revised. The reference to NTDs in the Introduction is now contextual (classifying scabies as an NTD) rather than implying a multi-NTD investigation. No NTD prevalence data beyond scabies were collected or reported in this study.

***R3 — Discussion: General — intellectual rigor and comparison with other studies***

**Reviewer comment:** *Generally, the discussion section lacks the intellectual rigor of comparison of findings with other studies; nor provides possible explanations for the observed findings.*

**Response:** We have substantially strengthened the intellectual rigor of the Discussion. The revised Discussion now systematically compares findings with studies from comparable settings, including: (1) the Alhaffar et al. 2025 MSF study from the same Cox’s Bazar setting, which reported similar age and sex distributions (Reference 8); (2) the Louka et al. 2022 Greece refugee study, which provides comparative proportional morbidity data from a European displacement context (Reference 7); (3) the Yitageasu et al. 2025 systematic review covering scabies burden across multiple displacement settings globally (Reference 5); and (4) studies from Australia, Fiji, the Solomon Islands, and the Netherlands for MDA effectiveness comparison (References 19, 36, 37). For each key finding — including the high burden among children, higher attack rate in the host community, and the durability of MDA impact — we have added explanatory sentences discussing possible mechanisms and contextualising the findings within the broader literature. The discussion of structural and social determinants (overcrowding, WASH constraints, caregiving patterns, language barriers, stigma) has been expanded to provide plausible explanations for the observed epidemiological patterns.

***R3 — Discussion, line 239: Turkish hospital comparison***

**Reviewer comment:** *Line 239: Are there comparative parameters of the Turkish hospital against which the data found in this study? If not, provide better reference for comparison.*

**Response:** The Turkish hospital reference (Turan et al.) has been replaced with a more directly comparable reference — the Alhaffar et al. 2025 study from the same Cox's Bazar setting (Reference 8) and the Louka et al. 2022 Greece refugee study (Reference 7), which provide appropriate comparative epidemiological parameters for our findings.

***R3 — Discussion, line 250–252: rationale for women's distribution***

**Reviewer comment:** *Lines 250–252: This proposed rationale is simply not good enough to explain the variations in scabies distribution in women and men.*

**Response:** The explanation for higher female attack rates has been substantially strengthened. The revised text now discusses: (1) women accompanying children to health facilities leading to overrepresentation in data; (2) increased caregiving close contact with infected children as a transmission risk (Reference 35, Mitchell et al. 2024); and (3) acknowledgment that biological susceptibility cannot be excluded and warrants further investigation.

***R3 — Discussion, line 254: "proportional" poorly used***

**Reviewer comment:** *Line 254: The use of the word "proportional" is poorly done here. Consider revising.*

**Response:** "Proportional" has been replaced with "relative contribution": "This relative contribution of scabies to overall morbidity in this setting substantially exceeds that reported in similar settings."

***R3 — Discussion, line 255: poor linking***

**Reviewer comment:** *Line 255: Poor linking of the context above (prevalence of scabies) and treatment.*

**Response:** The transition between the morbidity discussion and the treatment/resource burden has been revised with an explicit linking sentence: "Despite being a treatable condition with topical permethrin and oral ivermectin, the high volume of cases imposes a considerable burden on limited health system resources in this protracted humanitarian context."

***R3 — Discussion, lines 253–263: rewrite section***

**Reviewer comment:** *Lines 253–263: This section is poorly written with conversations all over the place. Consider completely rewriting this section.*

**Response:** The paragraph covering morbidity proportions, comparative settings, and treatment resource burden has been completely rewritten. The revised paragraph follows a clear logical structure: (1) morbidity contribution in IOM facilities; (2) comparison with similar settings; (3) resource burden implications; (4) need for cost-effective strategies.

***R3 — Discussion, line 277: "model" vs "findings"***

**Reviewer comment:** *Line 277: Be careful — the study reports "findings" and not "a model"! Please revise.*

**Response:** Corrected. All instances of "model" referring to this study's outputs have been replaced with "findings" or "analysis results".

***R3 — Discussion, lines 278–280: strong suggestion not strong statement***

**Reviewer comment:** *Lines 278–280: This point is strong. But with the study's lack of potential risk factors and other social parameters, this statement cannot be strongly set. It may be better only as a strong suggestion.*

**Response:** We have revised this sentence to present it as a recommendation rather than a definitive finding: "These findings suggest that while MDA is effective in rapidly reducing transmission, sustained control will likely require addressing the broader contextual factors, such as overcrowding, living conditions, and hygiene — though the retrospective nature of this study does not allow us to quantify the relative contribution of these factors."

***R3 — Discussion, lines 272–289: rewrite for readability***

**Reviewer comment:** *Consider rewriting lines 272 to 289 for better readability, synchrony and clarity.*

**Response:** This entire section of the Discussion has been restructured and rewritten. The revised paragraphs follow a clear, sequential logic: (1) MDA effectiveness confirmed; (2) durability and rebound; (3) structural factors; (4) MDA cycle recommendations; (5) economic burden; (6) host community inclusion. Each paragraph now addresses a single focused point.

***R3 — Conclusion, line 309: delete "extremely"***

**Reviewer comment:** *Line 309: Delete "extremely".*

**Response:** "Extremely" has been deleted. The sentence now reads: "The study revealed a high burden of scabies among the Rohingya refugees and the adjacent host community."

***R3 — Conclusion: tense and syntax***

**Reviewer comment:** *Check syntax and tense. Reporting is done in the past tense.*

**Response:** All present-tense verbs in the Conclusion have been corrected to past tense. Revised: "The study found a high burden..." and "The study found higher vulnerability among children and women..."

***R3 — Tables and figures: reformatting***

**Reviewer comment:** *Tables need reformatting.*

**Response:** Tables 1 and 2 have been reformatted: consistent column widths, clear cell borders, aligned text, and improved spacing have been applied. Table headings and variable labels have been standardised throughout.

We believe the revised manuscript now fully addresses all concerns raised by the editor and all three reviewers. We look forward to your decision.

Yours sincerely,

**Charls Erik Halder**, on behalf of all co-authors

Migration Health Division, International Organization for Migration (IOM), Bangladesh

Email: cehalder@iom.int
