# Supplementary material for: Massive scabies outbreak in Rohingya refugee camps, Cox’s Bazar: Epidemiology and impact of a mass drug administration (MDA) campaign – A retrospective study
Source: PLoS Negl Trop Dis. 2026 Jul 1;20(7):e0013832. doi: 10.1371/journal.pntd.0013832 (PMC13349296; doi:10.1371/journal.pntd.0013832)
Supplement: Peer review history

# Peer review: Decision Letter 0

**DOI:** 10.1371/journal.pntd.0013832.r001

**Authors:** Adly Abd-Alla, Michael Marks

13 Mar 2026

PNTD-D-25-02182

MASSIVE SCABIES OUTBREAK IN ROHINGYA REFUGEE CAMPS, COX’S BAZAR: EPIDEMIOLOGY AND IMPACT OF A MASS DRUG ADMINISTRATION (MDA) CAMPAIGN – A RETROSPECTIVE STUDY

PLOS Neglected Tropical Diseases

Dear Dr. Halder,

Thank you for submitting your manuscript to PLOS Neglected Tropical Diseases. After careful consideration, we feel that it has merit but does not fully meet PLOS Neglected Tropical Diseases's publication criteria as it currently stands. Therefore, we invite you to submit a revised version of the manuscript that addresses the points raised during the review process.

Please submit your revised manuscript within by May 12 2026 11:59PM. If you will need more time than this to complete your revisions, please reply to this message or contact the journal office at plosntds@plos.org. When you're ready to submit your revision, log on to https://www.editorialmanager.com/pntd/ and select the 'Submissions Needing Revision' folder to locate your manuscript file.

Please include the following items when submitting your revised manuscript:

* A letter that responds to each point raised by the editor and reviewer(s). You should upload this letter as a separate file labeled 'Response to Reviewers'. This file does not need to include responses to any formatting updates and technical items listed in the 'Journal Requirements' section below.

* A marked-up copy of your manuscript that highlights changes made to the original version. You should upload this as a separate file labeled 'Revised Manuscript with Track Changes'.

* An unmarked version of your revised paper without tracked changes. You should upload this as a separate file labeled 'Manuscript'.

If you would like to make changes to your financial disclosure, competing interests statement, or data availability statement, please make these updates within the submission form at the time of resubmission. Guidelines for resubmitting your figure files are available below the reviewer comments at the end of this letter.

We look forward to receiving your revised manuscript.

Kind regards,

Michael Marks

Academic Editor

PLOS Neglected Tropical Diseases

Adly Abd-Alla

Section Editor

PLOS Neglected Tropical Diseases

Shaden Kamhawi

co-Editor-in-Chief

PLOS Neglected Tropical Diseases

orcid.org/0000-0003-4304-636XX

Paul Brindley

co-Editor-in-Chief

PLOS Neglected Tropical Diseases

orcid.org/0000-0003-1765-0002

**Additional Editor Comments:**

Additional comments from the editor:

This is clearly an important dataset but as currently presented the manuscript requires considerable work. I have given concrete suggestions below which should be considered alongside the reviewers comments.

1) The writing throughout would benefit from re-reading.

2) There is little evidence to support the suggestion (line 65) that access to water is important for scabies. Washing does not remove scabies mites. Overcrowding is a risk factor but the evidence linking this directly to water access is very poor.

3) The MDA is inadequately described. There is no discussion of how the denominator population was ascertained, how the MDA was actually conducted, the coverage achieved, the time frame over which it was conducted. Without this information it is not possible to accurately evaluate the impact of the intervention. This section in both the methods and the results requires substantial rewriting.

4) The statistical methodology of the ITS is not adequately described. An ITS can look for an immediate effect but also an alteration of the overall trend - it is unclear what was looked for, how the ITS was fitted.

5) The description of the statistival software is lacking - version numbers should be provided (NB R Studio is also just an interface, the analysis software is R).

6) Ethics - was the only ehtics from a local hospital? Presumably WHO and IOM have ethics committees who were involved in the design and evaluation of the MDA intervention.

7) How do you account for population movement in calculating your case rates? It seems like yo have taken a fixed population as the denominator but included all cases? This is going to over-estimate the number of cases per population.

8) In the results you present a geospatial analysis which was never described in the methods.

9) Simiiarluy there are several other analysis that are never mentioned in the methods (for example bacterial infections)

**Journal Requirements:**

1) Please ensure that the CRediT author contributions listed for every co-author are completed accurately and in full.

At this stage, the following Authors/Authors require contributions: Charls Erik Halder, Md Abeed Hasan, James Charles Okello, David Otieno, Emmanuel Roba Soma, Partha Pratim Das, Md Mostafizur Rahman, Md Atiquzzaman, Hamim Tassdik, Dickson Wafula Barasa, Julekha Tabassum Poly, Abdullah Al Mamun, Abu Toha Md. Rezuanul Haque Bhuiyan, and U Maung Prue. Please ensure that the full contributions of each author are acknowledged in the "Add/Edit/Remove Authors" section of our submission form.

The list of CRediT author contributions may be found here: https://journals.plos.org/plosntds/s/authorship#loc-author-contributions

2) Please provide an Author Summary. This should appear in your manuscript between the Abstract (if applicable) and the Introduction, and should be 150-200 words long. The aim should be to make your findings accessible to a wide audience that includes both scientists and non-scientists. Sample summaries can be found on our website under Submission Guidelines:

https://journals.plos.org/plosntds/s/submission-guidelines#loc-parts-of-a-submission

3) We have noticed that you have uploaded Supporting Information files, but you have not included a list of legends. Please add a full list of legends for your Supporting Information files after the references list.

4) Some material included in your submission may be copyrighted. According to PLOSu2019s copyright policy, authors who use figures or other material (e.g., graphics, clipart, maps) from another author or copyright holder must demonstrate or obtain permission to publish this material under the Creative Commons Attribution 4.0 International (CC BY 4.0) License used by PLOS journals. Please closely review the details of PLOSu2019s copyright requirements here: PLOS Licenses and Copyright. If you need to request permissions from a copyright holder, you may use PLOS's Copyright Content Permission form.

Please respond directly to this email and provide any known details concerning your material's license terms and permissions required for reuse, even if you have not yet obtained copyright permissions or are unsure of your material's copyright compatibility. Once you have responded and addressed all other outstanding technical requirements, you may resubmit your manuscript within Editorial Manager.

Potential Copyright Issues:

- Figure 1: Please (a) provide a direct link to the base layer of the map (i.e., the country or region border shape) and ensure this is also included in the figure legend; and (b) provide a link to the terms of use / license information for the base layer image or shapefile. We cannot publish proprietary or copyrighted maps (e.g. Google Maps, Mapquest) and the terms of use for your map base layer must be compatible with our CC BY 4.0 license.

Note: if you created the map in a software program like R or ArcGIS, please locate and indicate the source of the basemap shapefile onto which data has been plotted.

If your map was obtained from a copyrighted source please amend the figure so that the base map used is from an openly available source. Alternatively, please provide explicit written permission from the copyright holder granting you the right to publish the material under our CC BY 4.0 license.

If you are unsure whether you can use a map or not, please do reach out and we will be able to help you. The following websites are good examples of where you can source open access or public domain maps:

* U.S. Geological Survey (USGS) - All maps are in the public domain. (http://www.usgs.gov)

* PlaniGlobe - All maps are published under a Creative Commons license so please cite u201cPlaniGlobe, http://www.planiglobe.com, CC BY 2.0u201d in the image credit after the caption. (http://www.planiglobe.com/?lang=enl)

* Natural Earth - All maps are public domain. (http://www.naturalearthdata.com/about/terms-of-use/).

**Reviewers' Comments:**

Reviewer's Responses to Questions

**Key Review Criteria Required for Acceptance?**

As you describe the new analyses required for acceptance, please consider the following:

**Methods:**

-Are the objectives of the study clearly articulated with a clear testable hypothesis stated?

-Is the study design appropriate to address the stated objectives?

-Is the population clearly described and appropriate for the hypothesis being tested?

-Is the sample size sufficient to ensure adequate power to address the hypothesis being tested?

-Were correct statistical analysis used to support conclusions?

-Are there concerns about ethical or regulatory requirements being met?

Reviewer #1: All items related to methods are well-designed, well-defined, and well-described.

There are no ethical concerns

Reviewer #2: yes

Reviewer #3: (No Response)

**********

**Results:**

-Does the analysis presented match the analysis plan?

-Are the results clearly and completely presented?

-Are the figures (Tables, Images) of sufficient quality for clarity?

Reviewer #1: The analysis match the plan

Results are clearly presented

Figures are helpful and illustrative

Reviewer #2: yes

Reviewer #3: (No Response)

**********

**Conclusions:**

-Are the conclusions supported by the data presented?

-Are the limitations of analysis clearly described?

-Do the authors discuss how these data can be helpful to advance our understanding of the topic under study?

-Is public health relevance addressed?

Reviewer #1: Conclusions are supported by the data, limitations are clearly stated, the public relevance of the study is clear and well addressed and authors discuss the study helpfulness

Reviewer #2: Yes - broadly

Reviewer #3: (No Response)

**********

**Editorial and Data Presentation Modifications?**

Use this section for editorial suggestions as well as relatively minor modifications of existing data that would enhance clarity. If the only modifications needed are minor and/or editorial, you may wish to recommend “Minor Revision” or “Accept”.

Reviewer #1: Minor drawbacks that I consider worth improving.

1- In the introduction, I don't think it's necessary nor accurate to use the adjective “intricately” in: “which intricately burrow…”

2- The following sentence is confusing. It seems to say that the WHO recommends two annual rounds of IVM, which does not correspond to the reality or the reference they provide as support:

“According to the WHO, the MDA regimen includes a dose of ivermectin at 200 µg/kg body weight administered biannually 7-14 days apart.”

Perhaps the authors want to say: According to the WHO, the MDA regimen consists of two doses of oral ivermectin at 200 µg/kg body weight, with an interval of 7 to 14 days between doses.

Please check!

3- The following sentence may be misleading: “As identified in several studies in the study setting as well as similar refugee contexts worldwide, overcrowded living arrangements, poor hygiene, and lack of water and sanitation facilities are mostly responsible for the high disease burden of scabies in the refugee settings (8,10,17)”

There may be an irregular interpretation of the references provided. The lack of water and sanitation cannot increase the burden of scabies. I recommend avoiding the word "responsible", replacing it with “associated factors”

On the other hand, is reference 8 appropriate? Maybe the intention was to use the 9?

4- The traditional scabies approach is not based on case-by-case management, but rather on the management of each case and its contacts. “Case-by-case and its contacts” management may also be appropriate.

Reviewer #2: Thanks for the opportunity to review this revised manuscript analysing the impact of the MDA for scabies undertaken at the camps in Coxs Bazar

I hope the following relatively minor comments are helpful:

line 88 _ small typo – take out “to)

Line 127: “MSF” I don’t think you have previously explained this abbreviation.

Line 128 – do you know why permethrin was mainly used?

MDA: do you know the % reach of the MDA? Please check the reference link – I don’t think it works anymore

Line 186: sentence check- may need to insert “that”

Table 2: I think it would be more eye catching as a bar chart

Table 3: suggest your two variables are either: “ infected and not infected” or “uncomplicated and infected”

Discussion

Repetition of the Greece data sentence line 69 and 238 – suggest removing – you can keep the Turkey reference.

244- could it be that more children are symptomatic and prone to secondary infection which is why they are brought to the clinics and reflected in the morbidity data you are measuring – this would be similar to what has been observed in eg indigenous Australian data.

271: do you want to consider including the additional benefits of ivermectin: being effective against a range of NTDs?

280: you advise addressing social determinants of health but could also recommend regular cycles of MDA at 6-12 monthly to address incoming cases, and or the impracticalities of addressing WASH etc?

References in general: please check the links you have included as July 2025 is sometime ago and the one that I checked didnt work.

Reviewer #3: (No Response)

**********

**Summary and General Comments:**

Use this section to provide overall comments, discuss strengths/weaknesses of the study, novelty, significance, general execution and scholarship. You may also include additional comments for the author, including concerns about dual publication, research ethics, or publication ethics. If requesting major revision, please articulate the new experiments that are needed.

Reviewer #1: Thank you very much for the opportunity to review this article, which addresses a highly relevant topic for public health, and is very helpful in supporting similar outbreaks in similar environments.

This is a retrospective study, and its limitations are well stated. Even so, the design and structure are clear, and the conclusions reached are interesting and supported by the data.

I only have comments on minor drawbacks that I consider worth improving.

Reviewer #2: I know we are awaiting the WHO Program Managers Guide to scabies outbreaks - I don't think it is quite there yet but it was expected at the end of 2025 so should be available soon and would be a great reference to include.

Reviewer #3: General comments

While the authors address an important topic, several aspects of the manuscript require significant improvement.

• A good study with strong outputs relevant to clinical care and interventions for scabies such as MDAs in endemic areas.

• The writeup contains a number of statements that are poorly referenced or not referenced at all.

• Authors should therefore consider rewriting – especially introduction and discussion portions – of the manuscript.

• Authors should opt for simpler clearer language and writing.

• The outcome of this study exposes the crucial need for the incorporation of behavioural, attitudinal and socioeconomic factors that cause infectious diseases into studies such as this.

Abstract/Summary

• Background:

o Satisfactory

o It may be important to state here the body responsible for the MDA campaign (i.e., authors? MoH?)

• Objectives: Consider rewriting sentence 1 (line 29): “The objectives of the study were: a) to determine the epidemiological characteristics (i.e., magnitude, age-sex distribution and attack rate) of the scabies outbreak in the Rohingya refugee camps.

• Methodology: Some more details of the methods is required. What exact information/ details is included in the IOM data that was used? MDA is mentioned at the end of results but no detail is provided on the type of MDA or approach for the MDA. Tis should be included.

• Results:

o Line 40-41: % decrease could be presented to distinctively show the rate of decline of attacks.

o Line 43: Same comment as above on smaller ratios for better relativity.

• Conclusion:

o Line 49: …was an effective approach for a rapid and…

Introduction

o Line 57: check referencing style. Delete (WHO) or replace with the clear sentence: “according to the World Health Organization”

o Line 57: Sarcoptes scabei (italicize) as it is a scientific name

o Line 59: Provide relevant literature to support documented mode of transmission i.e. skin-to-skin contact

o Line 63: The word “confront” is poorly used in this context/ sentence. Revise.

o Prior to the introduction of Rohingya into the conversation (in line 67), it would be better to provide some context/introduction/background of the camp.

o Line 66-67: The context provided is overly stretched. For e.g., it is better written as: “Such prevalence of scabies is seen in the Rohingya refugee camps in Bangladesh and this could be attributed to...”

o Line 66-67: Provide relevant proof/reference that support the claims.

o Line 68-69: Ensure consistency in presentation of historical prevalence rates. Stick to percentages or cases per year.

o Line 70-75 could be moved to just before line 66 for better reading and context. As it is, the introduction section looks very jointed and reads poorly.

o Line 75-76 looks out of place due to poor structuring.

o Line 76-77: the sentences are beginning to repeat. This assertion is already made in line 66-67.

o Line 82-83, the word “globally” must be deleted. Also, consider rewriting for clarity.

o Move the content of lines 82 – 86 to after line 94. The former shows the study’s problem statement and subsequent justification and is better if presented after describing the MDA campaign which is the core focus of the study. Also, it links better with line 95.

o Line 89: state the country of the camps

o Line 100: Delete the phrase “in summary”. Be succinct.

o Line 106: In which jurisdiction(s) will these findings be most useful. Indicate.

Methodology

The criteria for clinical diagnosis of scabies and secondary bacterial infections should be clearly stated.

The methods section should be improved. It seems to me that cases were seen and managed individually over several years before a decision was made for MDA. Thus, the authors need to indicate clearly the point at which the threshold for MDA was met/ what guided the decision to undertake the MDA. Further details need to be provided on the planning and engagement processes that took place prior to implementation of the actual MDA. Was any listing done? What was the total targeted population for MDA? Which category of people delivered the MDA?

o Line 114: …at any/either one of the 35…

o Lines 115-117: Delete or use to supplement content in introduction

o Line 122: Presence of secondary infection? Doesn’t “detection” or “diagnosis” read better?

o Line 123: What does “centrally cleaned” mean? For emphasis, simplicity is better. Avoid redundancy of words.

o Line 125-128: Authors should state clearly what definitions were used for the clinical diagnosis of scabies.

o Line 139: “Therapeutics” is poorly used here. Authors should stick to the simple e.g., “medication”.

o Line 138-142 read more like logistics than a report of the reach and mode of conduct of the MDA

o Line 146: If respective catchment populations are to be used, they must be highlighted somewhere in the methods section.

o Line 157: Kindly report the versions and manufacturer(s)/origins of the statistical tools used.

• Results

o Table 1: Format text spaces in the table and entire table for easier reading and understanding.

o Line 186: …which was performed……………..

o Throughout section, consider the tense/ syntax of the sentences.

o Method of presentation of data in the context/section is poor. Provide clear, easily quantifiable, figures. Finer details can then be in methodology section.

o Line 203 – 204 not necessary as there is a section for reporting that outcome.

o While the presentation of the section of secondary bacterial infection is important, context must be provided on this manuscript. More context must be provided in the introduction section.

o Table 3: Same issue as with table 1.

o Table 3 has same challenges raised as Table 1 and 2

o Figure 1: this approach is not mentioned in the methods/ analysis

• Discussion

o Line 231: The use of the word “and adjacent host communities” suggests data was collected from these areas as well?

o Line 237: Was there an investigation on a number of NTDs or their prevalence and were these listed in the results section? If not, consider removing.

o Generally, the discussion section lacks the intellectual rigor of comparison of findings with other studies; nor provides possible explanations for the observed findings.

o Like 239: Are there comparative parameters of the Turkish hospital against which the data found in this study? If not, provide better reference for comparison.

o Line 250-252: This proposed rationale is simply not good enough to explain the variations in scabies distribution in women and men.

o Line 254: The use of the word “proportional” is poorly done here. Consider revising.

o Line 255: Poor linking of the context above (prevalence of scabies) and treatment.

o Line 253-263: This section is poorly written with the conversations being all over the place. The paragraph poorly attempts to speak on too many contexts at the same time. Authors should do well to be as succinct in their presentation as possible. Consider completely rewriting this section.

o Line 277: Be careful with the use of certain terminology. The study reports “findings” and not “a model”! Please revise.

o Line 278-280: This point is strong. But with the study’s lack of potential risks factors and other social parameters, this statement cannot be strongly set. It may better only be a strong suggestion.

o Again, consider rewriting lines 272 to 289 for better readability, synchrony and clarity.

• Conclusion

o Line 309: Delete “extremely”.

o Check syntax and tense. Reporting is done in the past tense

• Tables and figures

o Tables need reformatting.

**********

PLOS authors have the option to publish the peer review history of their article (what does this mean?). If published, this will include your full peer review and any attached files.

If you choose “no”, your identity will remain anonymous but your review may still be made public.

**Do you want your identity to be public for this peer review?**  For information about this choice, including consent withdrawal, please see our Privacy Policy.

Reviewer #1: No

Reviewer #2: **Yes:** Dr Lucinda Claire Fuller

Reviewer #3: No

[NOTE: If reviewer comments were submitted as an attachment file, they will be attached to this email and accessible via the submission site. Please log into your account, locate the manuscript record, and check for the action link "View Attachments". If this link does not appear, there are no attachment files.]

**Figure resubmission:**

While revising your submission, we strongly recommend that you use PLOS’s NAAS tool (https://ngplosjournals.pagemajik.ai/artanalysis) to test your figure files. NAAS can convert your figure files to the TIFF file type and meet basic requirements (such as print size, resolution), or provide you with a report on issues that do not meet our requirements and that NAAS cannot fix.

After uploading your figures to PLOS’s NAAS tool - https://ngplosjournals.pagemajik.ai/artanalysis, NAAS will process the files provided and display the results in the "Uploaded Files" section of the page as the processing is complete. If the uploaded figures meet our requirements (or NAAS is able to fix the files to meet our requirements), the figure will be marked as "fixed" above. If NAAS is unable to fix the files, a red "failed" label will appear above. When NAAS has confirmed that the figure files meet our requirements, please download the file via the download option, and include these NAAS processed figure files when submitting your revised manuscript.

**Reproducibility:**

To enhance the reproducibility of your results, we recommend that authors of applicable studies deposit laboratory protocols in protocols.io, where a protocol can be assigned its own identifier (DOI) such that it can be cited independently in the future. Additionally, PLOS ONE offers an option to publish peer-reviewed clinical study protocols. Read more information on sharing protocols at https://plos.org/protocols?utm_medium=editorial-email&utm_source=authorletters&utm_campaign=protocols

# Author comment: Author response to Decision Letter 1

**DOI:** 10.1371/journal.pntd.0013832.r002

31 Mar 2026

Attachment Submitted filename: Response_to_Reviewers_PNTD-D-25-02182_v3.docx

# Peer review: Decision Letter 1

**DOI:** 10.1371/journal.pntd.0013832.r003

**Authors:** Adly Abd-Alla, Michael Marks, Álvaro Acosta-Serrano, Michael Marks

14 May 2026

PNTD-D-25-02182R1

Massive scabies outbreak in Rohingya refugee camps, Cox's Bazar: Epidemiology and impact of a mass drug administration (MDA) campaign – a retrospective study

PLOS Neglected Tropical Diseases

Dear Dr. Halder,

Thank you for submitting your manuscript to PLOS Neglected Tropical Diseases. After careful consideration, we feel that it has merit but does not fully meet PLOS Neglected Tropical Diseases's publication criteria as it currently stands. Therefore, we invite you to submit a revised version of the manuscript that addresses the points raised during the review process.

Please submit your revised manuscript by Jun 13 2026 11:59PM. If you will need more time than this to complete your revisions, please reply to this message or contact the journal office at plosntds@plos.org. When you're ready to submit your revision, log on to https://www.editorialmanager.com/pntd/ and select the 'Submissions Needing Revision' folder to locate your manuscript file.

Please include the following items when submitting your revised manuscript:

* A letter that responds to each point raised by the editor and reviewer(s). You should upload this letter as a separate file labeled 'Response to Reviewers'. This file does not need to include responses to any formatting updates and technical items listed in the 'Journal Requirements' section below.

* A marked-up copy of your manuscript that highlights changes made to the original version. You should upload this as a separate file labeled 'Revised Manuscript with Track Changes'.

* An unmarked version of your revised paper without tracked changes. You should upload this as a separate file labeled 'Manuscript'.

If you would like to make changes to your financial disclosure, competing interests statement, or data availability statement, please make these updates within the submission form at the time of resubmission. Guidelines for resubmitting your figure files are available below the reviewer comments at the end of this letter.

We look forward to receiving your revised manuscript.

Kind regards,

Michael Marks

Academic Editor

PLOS Neglected Tropical Diseases

Adly Abd-Alla

Section Editor

PLOS Neglected Tropical Diseases

Shaden Kamhawi

co-Editor-in-Chief

PLOS Neglected Tropical Diseases

orcid.org/0000-0003-4304-636XX

Paul Brindley

co-Editor-in-Chief

PLOS Neglected Tropical Diseases

orcid.org/0000-0003-1765-0002

**Reviewers' comments:**

Reviewer's Responses to Questions

**Key Review Criteria Required for Acceptance?**

As you describe the new analyses required for acceptance, please consider the following:

**Methods**

-Are the objectives of the study clearly articulated with a clear testable hypothesis stated?

-Is the study design appropriate to address the stated objectives?

-Is the population clearly described and appropriate for the hypothesis being tested?

-Is the sample size sufficient to ensure adequate power to address the hypothesis being tested?

-Were correct statistical analysis used to support conclusions?

-Are there concerns about ethical or regulatory requirements being met?

Reviewer #1: No concerns about methods

Reviewer #2: All the questions above area addressed adequately

**********

**Results**

-Does the analysis presented match the analysis plan?

-Are the results clearly and completely presented?

-Are the figures (Tables, Images) of sufficient quality for clarity?

Reviewer #1: Analysis and results matched the plan are are clear and completely presented

Reviewer #2: It might be possible to reduce the number of figures

**********

**Conclusions**

-Are the conclusions supported by the data presented?

-Are the limitations of analysis clearly described?

-Do the authors discuss how these data can be helpful to advance our understanding of the topic under study?

-Is public health relevance addressed?

Reviewer #1: Yes

Reviewer #2: Conclusions are supported by the data presented and its contribution to ongoing understanding around mass scabies outbreaks is well presented.

**********

**Editorial and Data Presentation Modifications?**

Use this section for editorial suggestions as well as relatively minor modifications of existing data that would enhance clarity. If the only modifications needed are minor and/or editorial, you may wish to recommend “Minor Revision” or “Accept”.

Reviewer #1: (No Response)

Reviewer #2: I think it would be appropriate to include reference to the largest MDA published so far:

Enbiale W, Baynie TB, Ayalew A, Gebrehiwot T, Getanew T, Ayal A, Ayalew M, De Vries HJ, Takarinda K, Manzi M, Zachriah R. "Stopping the itch": mass drug administration for scabies outbreak control covered for over nine million people in Ethiopia. J Infect Dev Ctries. 2020 Jun 29;14(6.1):28S-35S. doi: 10.3855/jidc.11701. PMID: 32614793.

and to sign post the recently launched WHO "Control of Scabies; A guide for national program managers"

chrome-extension://efaidnbmnnnibpcajpcglclefindmkaj/ https://www.worldscabiesprogram.org/media/n2rnv4ei/control_of_scabies_20260410final-18.pdf

**********

**Summary and General Comments**

Use this section to provide overall comments, discuss strengths/weaknesses of the study, novelty, significance, general execution and scholarship. You may also include additional comments for the author, including concerns about dual publication, research ethics, or publication ethics. If requesting major revision, please articulate the new experiments that are needed.

Reviewer #1: The manuscript is improved, and suitable por publication

Reviewer #2: A useful review of the management of the massive outbreak in CB by the local teams. Given the very hot off the press WHO guide for program managers for Control of Scabies, you could sign post to this in your discussion/conclusion as this now provides useful direction that you may well have appreciated at the time of your interventions.

Also noting your large number of cases treated, aware that the Ethiopian experience which whilst was not in a displaced camp setting, was non the less impressive in its reach, I suggest referencing this (see above)

**********

PLOS authors have the option to publish the peer review history of their article (what does this mean?). If published, this will include your full peer review and any attached files.

If you choose “no”, your identity will remain anonymous but your review may still be made public.

**Do you want your identity to be public for this peer review?**  For information about this choice, including consent withdrawal, please see our Privacy Policy.

Reviewer #1: No

Reviewer #2: **Yes:** Dr Lucinda Claire Fuller

[NOTE: If reviewer comments were submitted as an attachment file, they will be attached to this email and accessible via the submission site. Please log into your account, locate the manuscript record, and check for the action link "View Attachments". If this link does not appear, there are no attachment files.]

**Figure resubmission:**

While revising your submission, we strongly recommend that you use PLOS’s NAAS tool (https://ngplosjournals.pagemajik.ai/artanalysis) to test your figure files. NAAS can convert your figure files to the TIFF file type and meet basic requirements (such as print size, resolution), or provide you with a report on issues that do not meet our requirements and that NAAS cannot fix.

After uploading your figures to PLOS’s NAAS tool - https://ngplosjournals.pagemajik.ai/artanalysis, NAAS will process the files provided and display the results in the "Uploaded Files" section of the page as the processing is complete. If the uploaded figures meet our requirements (or NAAS is able to fix the files to meet our requirements), the figure will be marked as "fixed" above. If NAAS is unable to fix the files, a red "failed" label will appear above. When NAAS has confirmed that the figure files meet our requirements, please download the file via the download option, and include these NAAS processed figure files when submitting your revised manuscript.

**Reproducibility:**

To enhance the reproducibility of your results, we recommend that authors of applicable studies deposit laboratory protocols in protocols.io, where a protocol can be assigned its own identifier (DOI) such that it can be cited independently in the future. Additionally, PLOS ONE offers an option to publish peer-reviewed clinical study protocols. Read more information on sharing protocols at https://plos.org/protocols?utm_medium=editorial-email&utm_source=authorletters&utm_campaign=protocols

# Author comment: Author response to Decision Letter 2

**DOI:** 10.1371/journal.pntd.0013832.r004

17 May 2026

Attachment Submitted filename: Response to reviewers.docx

# Editor report: Decision Letter 2

**DOI:** 10.1371/journal.pntd.0013832.r005

**Authors:** Adly Abd-Alla, Michael Marks, Álvaro Acosta-Serrano, Michael Marks, Álvaro Acosta-Serrano

17 Jun 2026

Dear Dr. Halder,

We are pleased to inform you that your manuscript 'Massive scabies outbreak in Rohingya refugee camps, Cox's Bazar: Epidemiology and impact of a mass drug administration (MDA) campaign – a retrospective study' has been provisionally accepted for publication in PLOS Neglected Tropical Diseases.

Before your manuscript can be formally accepted you will need to complete some formatting changes, which you will receive in a follow up email. A member of our team will be in touch with a set of requests.

Please note that your manuscript will not be scheduled for publication until you have made the required changes, so a swift response is appreciated.

IMPORTANT: The editorial review process is now complete. PLOS will only permit corrections to spelling, formatting or significant scientific errors from this point onwards. Requests for major changes, or any which affect the scientific understanding of your work, will cause delays to the publication date of your manuscript.

Should you, your institution's press office or the journal office choose to press release your paper, you will automatically be opted out of early publication. We ask that you notify us now if you or your institution is planning to press release the article. All press must be co-ordinated with PLOS.

Thank you again for supporting Open Access publishing; we are looking forward to publishing your work in PLOS Neglected Tropical Diseases.

Best regards,

Álvaro Acosta-Serrano

Section Editor

PLOS Neglected Tropical Diseases

Shaden Kamhawi

co-Editor-in-Chief

PLOS Neglected Tropical Diseases

orcid.org/0000-0003-4304-636XX

Paul Brindley

co-Editor-in-Chief

PLOS Neglected Tropical Diseases

orcid.org/0000-0003-1765-0002

***********************************************************

p.p1 {margin: 0.0px 0.0px 0.0px 0.0px; line-height: 16.0px; font: 14.0px Arial; color: #323333; -webkit-text-stroke: #323333}span.s1 {font-kerning: none

# Editor report: Acceptance letter

**DOI:** 10.1371/journal.pntd.0013832.r006

**Authors:** Adly Abd-Alla, Michael Marks, Álvaro Acosta-Serrano, Michael Marks, Álvaro Acosta-Serrano

Dear Dr. Halder,

We are delighted to inform you that your manuscript, "Massive scabies outbreak in Rohingya refugee camps, Cox's Bazar: Epidemiology and impact of a mass drug administration (MDA) campaign – a retrospective study," has been formally accepted for publication in PLOS Neglected Tropical Diseases.

We have now passed your article onto the PLOS Production Department who will complete the rest of the publication process. All authors will receive a confirmation email upon publication.

The corresponding author will soon be receiving a typeset proof for review, to ensure errors have not been introduced during production. Please review the PDF proof of your manuscript carefully, as this is the last chance to correct any scientific or type-setting errors. Please note that major changes, or those which affect the scientific understanding of the work, will likely cause delays to the publication date of your manuscript. Note: Proofs for Front Matter articles (Editorial, Viewpoint, Symposium, Review, etc...) are generated on a different schedule and may not be made available as quickly.

Soon after your final files are uploaded, the early version of your manuscript will be published online unless you opted out of this process. The date of the early version will be your article's publication date. The final article will be published to the same URL, and all versions of the paper will be accessible to readers.

For Research Articles, you will receive an invoice from PLOS for your publication fee after your manuscript has reached the completed accept phase. If you receive an email requesting payment before acceptance or for any other service, this may be a phishing scheme. Learn how to identify phishing emails and protect your accounts at https://explore.plos.org/phishing.

Thank you again for supporting open-access publishing; we are looking forward to publishing your work in PLOS Neglected Tropical Diseases.

Best regards,

Shaden Kamhawi

co-Editor-in-Chief

PLOS Neglected Tropical Diseases

Paul Brindley

co-Editor-in-Chief

PLOS Neglected Tropical Diseases
